# Defining the phospho-adhesome through the phosphoproteomic analysis of integrin signalling

**DOI:** 10.1038/ncomms7265

**Published:** 2015-02-13

**Authors:** Joseph Robertson, Guillaume Jacquemet, Adam Byron, Matthew C. Jones, Stacey Warwood, Julian N. Selley, David Knight, Jonathan D. Humphries, Martin J. Humphries

**Affiliations:** 1 Wellcome Trust Centre for Cell-Matrix Research, Faculty of Life Sciences, University of Manchester, Manchester M13 9PT, UK; 2 Biological Mass Spectrometry Core Facility, Faculty of Life Sciences, University of Manchester, Manchester M13 9PT, UK

## Abstract

Cell–extracellular matrix (ECM) adhesion is a fundamental requirement for multicellular existence due to roles in positioning, proliferation and differentiation. Phosphorylation plays a major role in adhesion signalling; however, a full understanding of the phosphorylation events that occur at sites of adhesion is lacking. Here we report a proteomic and phosphoproteomic analysis of adhesion complexes isolated from cells spread on fibronectin. We identify 1,174 proteins, 499 of which are phosphorylated (1,109 phosphorylation sites), including both well-characterized and novel adhesion-regulated phosphorylation events. Immunoblotting suggests that two classes of phosphorylated residues are found at adhesion sites—those induced by adhesion and those constitutively phosphorylated but recruited in response to adhesion. Kinase prediction analysis identifies novel kinases with putative roles in adhesion signalling including CDK1, inhibition of which reduces adhesion complex formation. This phospho-adhesome data set constitutes a valuable resource to improve our understanding of the signalling mechanisms through which cell–ECM interactions control cell behaviour.

## Abstract (web summary)

Protein phosphorylation is known to play an important role in cell adhesion signalling. Robertson *et al*. present a proteomic resource mapping the phosphorylation states of proteins isolated from adhesion complexes and, taking advantage of this data set, show that the cell cycle kinase CDK1 may influence cell adhesion.

## Graphical abstract

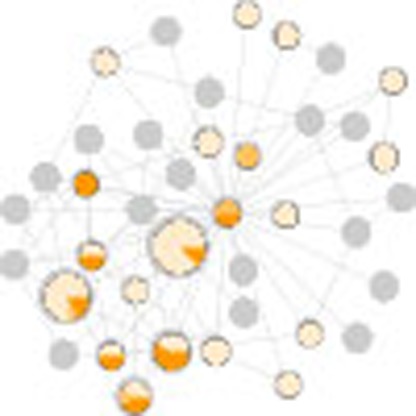

Cellular adhesion to the extracellular matrix (ECM), mediated by adhesion receptors such as integrins and syndecans, initiates or modulates signalling pathways that control a range of cell functions including proliferation, survival, differentiation and migration^1^,^2^,^3^. Accordingly, adhesion plays an important role in processes such as development, wound healing and inflammation, while aberrant adhesion is associated with diseases such as immunodeficiency, bleeding disorders and cancer^4^,^5^. Adhesion signalling is orchestrated through large and diverse multiprotein complexes that are recruited to sites of cell–ECM engagement (adhesion complexes). Literature-based analyses of adhesion complexes have revealed highly connected networks of at least 180 distinct components (collectively termed the integrin ‘adhesome’)^5^,^6^,^7^, including cytoskeletal linkers and regulators, and enzymes involved in a plethora of cell signalling pathways. More recently, the development of methodologies for the isolation of adhesion complexes has enabled mass spectrometry (MS)-based proteomic profiling of their molecular composition^8^,^9^,^10^,^11^,^12^. Such proteomic approaches have begun to reveal the true diversity and complexity of the adhesome, as well as provide insights into how adhesion complex composition varies in an integrin heterodimer- and tension-dependent manner^8^,^9^,^10^,^11^,^12^,^13^.

Reversible phosphorylation of serine, threonine and tyrosine residues is a prominent signalling mechanism to enable spatial and temporal regulation of the activation states, conformations or binding interactions of proteins and thereby regulate diverse downstream effects^14^. Phosphorylation is postulated to play an important and widespread role in signalling within adhesion sites^6^. For example, a large number of kinases and phosphatases are recruited to adhesion sites, and several of these enzymes are highly connected within the adhesome network (forming putative ‘hubs’)^7^. Furthermore, a number of tyrosine phosphorylation events on adhesome proteins such as FAK, Src, paxillin and p130Cas are induced by adhesion and play important functional roles in integrin signalling^15^,^16^,^17^,^18^. Indeed, the immunostaining of ECM-adherent cells using generic anti-phosphotyrosine antibodies has revealed that, in general, adhesion sites possess high levels of tyrosine-phosphorylated proteins^18^,^19^,^20^,^21^. Although systems-level analyses of adhesion-induced phosphorylation events have been performed^11^,^22^, a detailed catalogue of the phosphorylation sites found specifically on proteins within adhesion complexes is lacking. Here we have addressed this deficit by applying a combination of proteomic and phosphoproteomic methodologies to the analysis of isolated adhesion complexes, and thereby have generated data sets that can be utilized to provide novel insights into the mechanisms of adhesion signalling. For example, by validating a number of the identified phosphorylation sites by immunoblotting, we have identified distinct populations of adhesion-induced and adhesion-independent phosphorylation sites that are recruited to adhesion complexes. Moreover, these data sets may have wide-ranging implications for other areas of biological research, highlighting potential novel links with cellular adhesion. To this end, we have identified a number of protein kinases with putative novel roles in adhesion signalling, and shown that one such kinase, cyclin-dependent kinase 1 (CDK1), has a role in regulating adhesion complex formation.

## Results

### Proteomic and phosphoproteomic analysis of adhesion complexes

**Figure 1:**
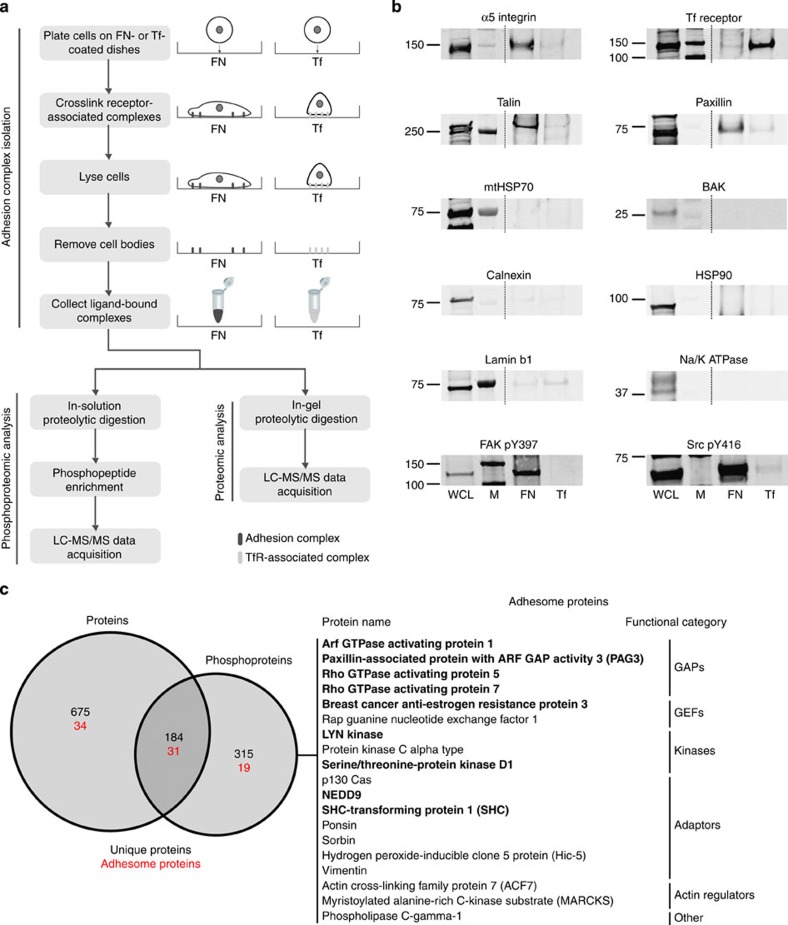
Combined proteomic and phosphoproteomic analysis of isolated adhesion complexes. (**a**) Schematic workflow for the isolation and proteomic/phosphoproteomic analysis of adhesion complexes. Cells were allowed to spread on FN or, as a control, Tf and complexes were isolated by a combination of crosslinking, cell lysis and a high-pressure wash to remove cell bodies. Collected complexes were analysed using either a proteomic or phosphoproteomic workflow, after which the FN-specific proteins and phosphoproteins were identified by performing a subtractive comparison with controls. (**b**) Immunoblot analysis of complexes isolated from cells spread on FN and Tf, as well as the WCLs of cells spread on FN. M, MW markers (kDa; values displayed to the left of each blot). Dashed lines indicate where images have been cropped for display purposes. (**c**) A Venn diagram showing the overlap between the FN-specific proteins (left circle) and phosphoproteins (right circle) identified by proteomic and phosphoproteomic analyses of isolated complexes, respectively. In addition to the total number of proteins (black text), the number of adhesome proteins identified in each data set is also displayed (red text). To the right of the panel, all 19 adhesome components identified exclusively by the phosphoproteomic analysis are displayed. Proteins in bold text were not identified by any other proteomic analyses of isolated FN-induced adhesion complexes.

To define the protein and phosphoprotein composition of adhesion sites, a combined proteomic and phosphoproteomic workflow was used to analyse adhesion complexes isolated from cells spread on the ECM protein fibronectin (FN; Fig. 1a). To aid the identification of specific, integrin-associated proteins, complexes were also isolated from cells spread on the control substrate transferrin (Tf) and analysed using the same workflow. Immunoblotting of isolated complexes demonstrated that α5 integrin (a subunit of the FN-binding integrin α5β1) and the well-characterized adhesion complex proteins paxillin and talin were enriched in FN-induced adhesion complexes, while the Tf receptor (TfR) was enriched in Tf-induced protein complexes (Fig. 1b). Non-adhesion-associated proteins (from various cellular compartments) such as mitochondrial heat shock protein (HSP)-70, HSP90 (cytoplasm), Na^+^/K^+^-ATPase (plasma membrane), TfR (plasma membrane and endosome), BAK (mitochondria), calnexin (endoplasmic reticulum) and lamin B1 (nucleus) were not detected to a significant level in the complexes isolated from cells spread on either ligand (Fig. 1b). Furthermore, the well-characterized adhesion-induced phosphorylation sites on FAK and Src (pY397 and pY416, respectively) were specifically detected in FN-induced adhesion complexes (Fig. 1b). Together these data demonstrate that the isolation method specifically enriched and retained adhesion complex proteins, including those containing phosphorylation sites that were previously reported to be phosphorylated in response to integrin-mediated adhesion to the ECM.

Proteomic and phosphoproteomic analyses identified 859 proteins and 499 phosphoproteins (containing a total of 1,109 phosphorylation sites) that were specifically detected in FN-induced adhesion complexes (following subtractive comparisons with controls; Supplementary Data 1–4). Compared with previous proteomic analyses of adhesion complexes isolated from cells spread on FN, the proteomic data set collected here contained a similar number of proteins and displayed a similar level of detection of previously defined adhesome components (65; 36% of the adhesome)^13^. Many well-characterized adhesion complex proteins such as talin, vinculin, FAK and paxillin were detected in the proteomic data set, as well as the two FN-binding integrin heterodimers α5β1 and αvβ3. The phosphoproteomic data set, however, contained a large number of proteins (315) that were not identified by the proteomic analysis. Thus, the total number of identified adhesion complex proteins was substantially increased by merging the proteomic and phosphoproteomic data sets (from 859 to 1,174; an increase of 36%; Fig. 1c). Notably, 19 of the proteins identified only in the phosphoproteomic analysis were previously defined adhesome components, providing confidence that the extra proteins identified by phosphoproteomic analysis were genuine adhesion complex proteins. Interestingly, 10 of these adhesome proteins have not been identified in any other published adhesion complex proteomic analyses^8^,^9^,^10^,^11^,^12^ (Fig. 1c). These findings indicate that performing a combined proteomic and phosphoproteomic analysis of isolated adhesion complexes can generate a more comprehensive and representative catalogue of components than is possible by proteomic analysis alone.

### Comparing adhesion complex and whole cell phosphoproteomes

To assess the specificity of adhesion-associated phosphoprotein detection from phosphoproteomic analysis of isolated adhesion complexes, gene ontology (GO) enrichment analysis of the 499 phosphoproteins identified from isolated adhesion complexes was performed. This revealed a significant enrichment of biological process (BP) terms associated with adhesion biology (such as ‘regulation of cytoskeleton organization’ and ‘regulation of small GTPase-mediated signal transduction’; Supplementary Fig. 1a and Supplementary Data 5). As whole-cell lysates (WCLs) are conventionally used for phosphoproteomic analyses, we also performed a phosphoproteomic analysis of the WCLs of cells spread on FN and searched the resulting data set for the enrichment of BP terms. Compared with isolated adhesion complexes, analysis of the WCL data set (735 phosphoproteins) revealed a very different set of enriched BP terms (Supplementary Fig. 1b), with many of the highest ranking BP terms related to RNA processing or the cell nucleus (for example, ‘chromosome organization’ and ‘DNA metabolic process’; Supplementary Fig. 1c and Supplementary Data 6). GO enrichment analyses of other WCL data sets collected in phosphoproteomic studies (involving different cell types, experimental conditions and total numbers of phosphoproteins^23^,^24^) revealed an enrichment of similar BP terms to those enriched in the WCL data set collected in the present study (for example, the highest ranking term was ‘RNA processing’ for all data sets; Supplementary Fig. 1c; Supplementary Data 6–8). Collectively, these data indicate that a substantial proportion of the most abundant phosphorylation sites in cells belong to proteins involved in RNA-related cellular processes. Notably, terms enriched in WCL data sets relating to RNA processing or the cell nucleus (Supplementary Fig. 1c) were not enriched in the isolated adhesion complex data set collected in this study (Supplementary Data 5). This indicates that phosphoproteins identified from isolated adhesion complexes are specific to the adhesion machinery of the cell, and that the bulk of the cellular phosphoproteome had been removed on adhesion complex isolation.

The specificity afforded by analysing isolated adhesion complexes enabled the detection of a number of phosphorylation sites that are known to be upregulated by adhesion to the ECM. These included sites on FAK (pY397, pY576 and pY577), paxillin (pY118) and six phosphotyrosine residues within the substrate domain of p130Cas, all of which have been shown to be stimulated by cell spreading on ECM ligands^25^,^26^,^27^. In addition, the phosphorylation sites were detected from other well-defined adhesion complex proteins that are less well documented to be phosphorylated in an adhesion-dependent manner. These included β1 integrin (pT777 and pY783), β3 integrin (pY773 and pY785), talin-1 (pY127, pT144, pS425, pY436 and pS2040), vinculin (pS290), kindlin-2 (pS159, pY179 and pS181), p190RhoGAP (pS589 and pY1105) and GIT1 (pY383, pS385, pS592, pS596 and pT601). It is notable that the majority of these sites were only detected through analysis of isolated adhesion complexes (none of the well-characterized adhesion-regulated phosphorylation sites on FAK, paxillin or p130Cas were detected through phosphoproteomic analysis of the WCLs of cells spread on FN performed in this study). These phosphorylation sites were also not detected in two large-scale adhesion-based phosphoproteomic studies in which the WCLs of cells spread on FN or collagen were analysed^11^,^22^. These observations indicate that many phosphorylation sites on adhesion complex proteins are of low abundance in the context of the whole-cell phosphoproteome, and are therefore difficult to detect within complex WCLs. As a result, the phosphoproteomic analysis of isolated adhesion complexes performed here enabled a more detailed coverage of phosphorylation events from adhesion structures.

### Creation of a phospho-adhesome network

**Figure 2:**
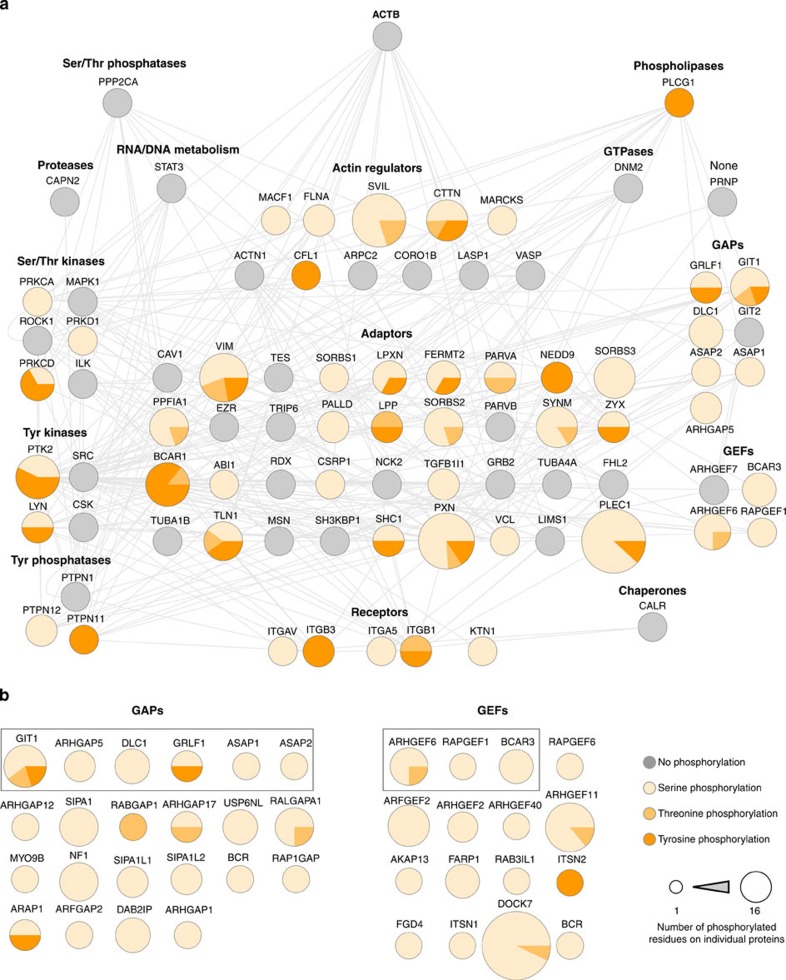
The FN-induced phospho-adhesome. (**a**) Adhesome components^6^ identified from the combined proteomic and phosphoproteomic analysis of isolated adhesion complexes were mapped onto a human PPI network and organized according to functional category. Grey nodes represent proteins identified by the proteomic analysis but not the phosphoproteomic analysis (no phosphorylation). All other nodes represent proteins identified by the phosphoproteomic analysis and are displayed as pie charts showing the relative levels of phosphorylated serine, threonine and tyrosine residues on each protein. These nodes are also displayed in different sizes to reflect the total number of phosphorylated residues identified from each protein. (**b**) All phosphorylated GEFs and GAPs identified from isolated adhesion complexes are displayed. Nodes surrounded by a grey box represent adhesome components.

To view the current literature-based depiction of the adhesome network^7^ in the context of the regulatory role of phosphorylation, a protein–protein interaction (PPI) network was constructed for all detected adhesome proteins. The network was arranged according to the functional subnetworks of the adhesome and overlaid with information on the number of phosphorylation sites detected in each protein (Fig. 2a). This revealed that phosphorylation is highly abundant among the identified adhesome components—of the 84 adhesome proteins that were detected, 50 were phosphorylated (on a total of 164 phosphorylation sites; the total number of phosphorylation sites per protein ranged from one to sixteen). In total, nine of the detected adhesome subnets contained at least one phosphoprotein, consistent with a widespread role for phosphorylation in the regulation of adhesion complex proteins. Furthermore, it is noteworthy that FAK and paxillin, two of the most highly connected proteins in the PPI network (thus representing putative essential ‘hubs’), were also two of the most highly phosphorylated adhesome proteins, highlighting the potentially fundamental role for phosphorylation in the regulation of protein interactions within adhesion sites. The scaffolding network of the adhesome (receptors, adaptors, actin regulators and actin) appeared, in general, to be highly regulated by phosphorylation, although distinct patterns were observed for individual subnets. For example, all five of the identified receptors were phosphorylated, although these proteins possessed low numbers of phosphorylated residues (≤2). Unlike the receptors, the identified adaptors and actin regulators displayed a large variation in the numbers of detected phosphorylation sites. Approximately half were not phosphorylated; however, eight of the nine most highly phosphorylated adhesome proteins were adaptors and actin regulators (six adaptors and two actin regulators, all of which were detected with six or more phosphorylation sites).

Outside of the scaffolding proteins, a large proportion of the identified guanine nucleotide exchange factors (GEFs) and GTPase-activating proteins (GAPs) from the adhesome were phosphorylated (Fig. 2a). The GAPs and GEFs appeared to be predominantly regulated by serine/threonine rather than tyrosine phosphorylation, with the GEFs subnet being the only subnet from which no tyrosine phosphorylation sites were detected. Interestingly, this pattern of phosphorylation was observed not only for the detected adhesome proteins, but for the whole data set: the majority of phosphorylation sites detected from all identified GEFs and GAPs were serines and threonines, with very few phosphorylated tyrosine residues (Fig. 2b). This was especially the case for the GEFs, from which only one tyrosine residue was detected on intersectin-2, which also functions as an adaptor. These data confirm and extend one of the observations of the literature-based analysis of adhesome components, which highlighted that GEFs are primarily regulated by serine/threonine kinases^7^. Conversely, the literature-based adhesome analysis also indicated that GAPs are primarily regulated by tyrosine kinases^7^, which is not consistent with the relatively high levels of serine and threonine phosphorylation detected on GAPs in this study. Thus, the data collected here both corroborate the previous observation from the literature that serine/threonine kinases play a prominent role in the regulation of GEFs, as well as provide novel insights into adhesion signalling that were not possible through extensive mining of the literature.

### Analysis of β1 integrin-proximal phosphorylation events

To provide a wider view of the proteins and phosphoproteins identified from adhesion complexes, a PPI network was constructed for proteins within one- and two-binding interactions of integrin β1 (Supplementary Fig. 2). This network was substantially larger and more complex than the adhesome component network: 369 proteins and phosphoproteins (31% of the total protein and phosphoprotein data set) were within the 2-hop neighbourhood of β1 integrin (39 and 330 within one and two binding interactions, respectively). Of the 369 proteins in the 2-hop neighbourhood, 168 were phosphorylated (46%). These included a number of proteins that do not have well-characterized roles in integrin-mediated signalling.

**Figure 3:**
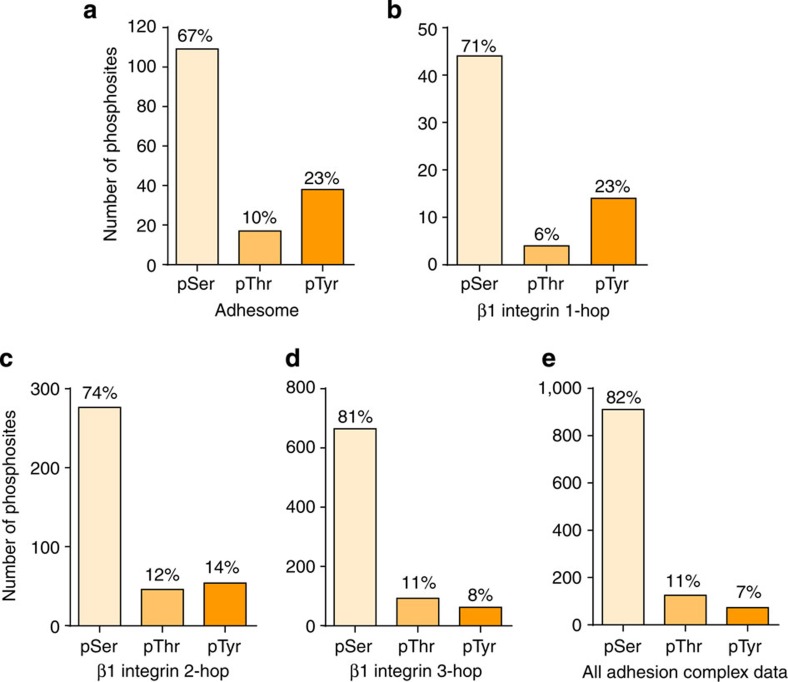
Serine, threonine and tyrosine phosphorylation within adhesion complexes. Numbers of serine, threonine and tyrosine phosphorylation sites detected from different populations of the proteins identified from isolated adhesion complexes are displayed. The displayed populations are: adhesome components (**a**), and proteins within one interaction (1-hop; **b**), two interactions (2-hop; **c**) and three interactions (3-hop; **d**) of β1 integrin. In addition, numbers of serine, threonine and tyrosine phosphorylation sites detected from all identified adhesion complex proteins are also displayed (**e**). For each population of proteins, percentages of serine, threonine and tyrosine phosphorylation are displayed above the corresponding bars.

The percentages of tyrosine phosphorylation for both the adhesome components (Fig. 2a) and the proteins in the β1 integrin one-hop neighbourhood (Supplementary Fig. 2) were 23% (Fig. 3a,b): a much higher percentage than that observed for the whole data set (7%; Fig. 3e). In fact, the percentage of tyrosine-phosphorylated residues on adhesion complex proteins correlated with proximity to β1 integrin (Fig. 3a–d). However, it is noteworthy that the proportion of tyrosine phosphorylation observed for the whole data set (7%; Fig. 3e) is also higher than that typically observed in larger-scale cell or tissue lysate-based phosphoproteomic studies (~2%) (refs 28, 29). These observations suggest that, compared with the whole-cell phosphoproteome, adhesion complexes are generally enriched for phosphotyrosine, and that this phosphotyrosine enrichment is more prominent for β1 integrin-proximal proteins.

### Validation of adhesion complex-specific phosphorylation sites

**Figure 4:**
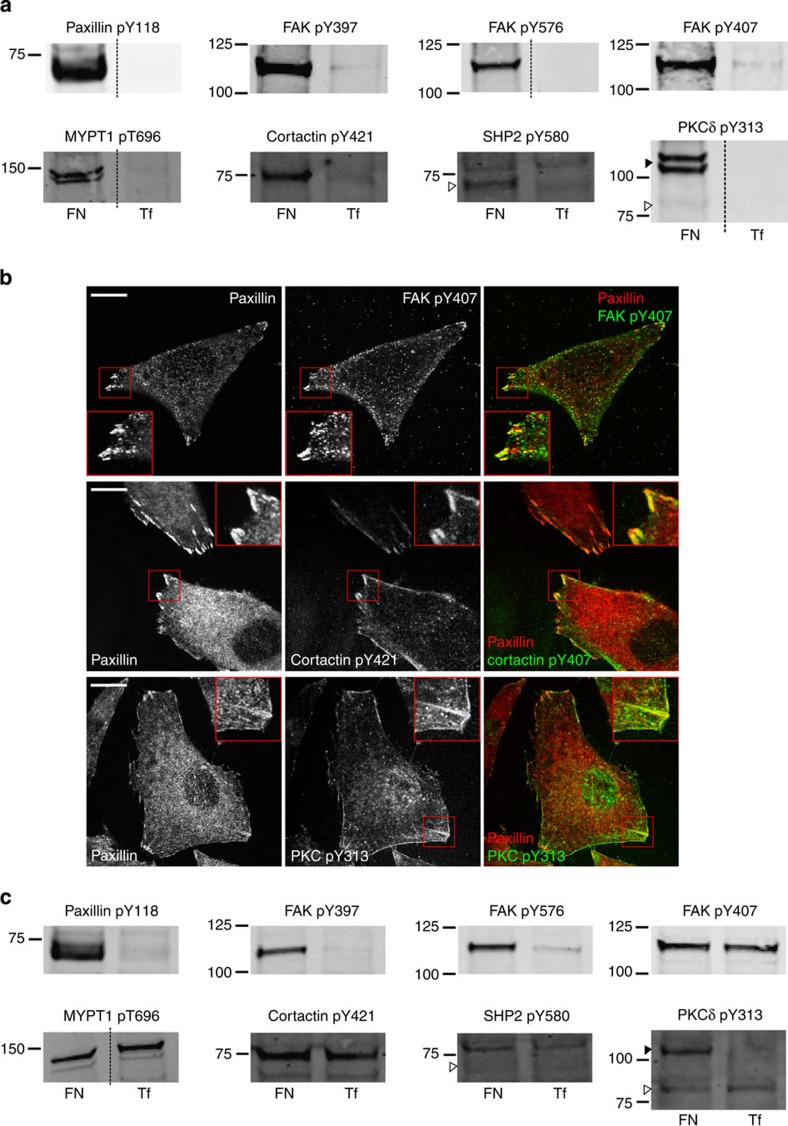
Validation of adhesion complex-localized phosphorylation sites. (**a**) Immunoblot analyses of the protein complexes isolated from cells spread on FN or Tf for 120 min. Samples were probed using phosphospecific antibodies targeting: paxillin pTyr118; FAK pTyr397, pTyr576 and pTyr407; MYPT1 pThr696; cortactin pTyr421; SHP2 pTyr580; and PKCδ pTyr313. (**b**) Immunofluorescence images of cells spread on FN for 120 min. Cells were stained using the phosphospecific antibodies FAK pTyr407, cortactin pTyr421 and PKCδ pTyr313 (all green). In each case, cells were also stained using an antibody targeting the focal adhesion marker paxillin (red). Scale bar, 20 μm. (**c**) Immunoblot analyses of the WCLs of cells spread on FN or Tf for 120 min. Samples were probed using the same antibodies as used to probe isolated complexes in **a**. Dashed lines in **a**,**c** indicate where the images have been cropped for display purposes. White arrows to left of blots highlight the expected MWs of the corresponding proteins. Black arrows to the left of blots highlight a putative high MW isoform of PKCδ.

To interrogate and validate the phosphoproteomic data further, immunoblotting and immunofluorescence analyses were performed using antibodies targeting both well-characterized adhesion-regulated phosphorylation sites and phosphorylation sites that are not known to be upregulated by adhesion to FN (Fig. 4). Consistent with MS data, immunoblotting analyses demonstrated that phosphorylation sites on paxillin (pY118), FAK (pY397, pTyr407 and pY576), MYPT1 (pT696), cortactin (pY421) and SHP2 (pY580) were all enriched to isolated adhesion complexes compared with controls (Fig. 4a). Probing for PKCδ pY313 produced a faint band that was specific to adhesion complexes and matched with the molecular weight (MW) of PKCδ (82 kDa; white arrow); however, an intense, higher-MW adhesion complex-specific double band (between 100 and 150 kDa; black arrow) was also observed. These data are consistent with a previous study reporting a 130-kDa isoform of PKCδ^30^, and suggest that both isoforms of the protein are recruited to adhesion complexes, with the higher-MW isoform being present in particularly high abundance.

Immunofluorescence analyses were consistent with both MS data and immunoblotting, demonstrating that the phosphorylation sites FAK pY407, cortactin pY421 and PKCδ pY313 co-localized with paxillin (a marker for adhesion complexes) in cells adherent to FN (Fig. 4b). Collectively, these data validated several components of the phosphoproteomic data set collected in this study and indicated that the data set as a whole represents genuine phosphorylation events in the adhesion complex signalling machinery. It was therefore anticipated that these phosphorylation sites would be regulated by adhesion and that this regulation would be detectable by immunoblotting of the lysates of ECM-engaged cells. To test this hypothesis, WCLs of cells spread on FN or Tf were immunoblotted using the same antibodies as used for MS data validation (Fig. 4c). As expected, the well-documented adhesion-regulated phosphorylation site paxillin pY118 was detected at much higher levels from the lysates of cells spread on FN compared with controls. Similar levels of adhesion-dependent upregulation were observed for two other phosphorylation sites from well-characterized components of integrin signalling pathways: FAK pY397 and pY576. Conversely, FAK pY407 was detected at similar levels from WCLs of cells spread on FN and Tf. Thus, although FAK pY407 was clearly present within adhesion complexes (Fig. 4a,b), the abundance of this phosphorylation site in cells did not appear to be substantially upregulated by cellular adhesion to FN (Fig. 4c). Intriguingly, other phosphorylation sites that were detected specifically from adhesion complexes showed similar patterns of detection to FAK pY407 (for example, MYPT1 pT696 and cortactin pY421; Fig. 4c).

Unlike immunoblot analysis of isolated adhesion complexes, a SHP2 pY580 band could not be detected from cell lysates (Fig. 4a,c). This suggests that the level of SHP2 pY580 in whole cells is too low to be detected by immunoblotting, but specific isolation (and therefore enrichment) of adhesion complex proteins can facilitate detection by both immunoblotting and MS. Immunoblotting for PKCδ pY313 again produced two bands of distinct MW, presumably representing two different isoforms. The band that matched with the expected MW of PKCδ (82 kDa) was detected at low and equal levels in the lysates of cells spread on FN and Tf (Fig. 4c; white arrow); however, the putative higher-MW isoform was present at much higher levels in the lysates of adherent cells compared with controls (Fig. 4c; black arrow). This indicates that, like pY118 of paxillin and pY397 and pY576 of FAK, pY313 of the higher-MW PKCδ isoform was upregulated by cell spreading on FN.

Collectively, these data validate the phosphoproteomic data retrieved from isolated adhesion complexes in this study, and also reveal that two populations of phosphorylation sites are present within adhesion sites: one that is substantially upregulated in response to adhesion, and one that appears to be constitutively phosphorylated in an adhesion-independent manner.

### Predicted kinase activities in adhesion-mediated signalling

**Figure 5:**
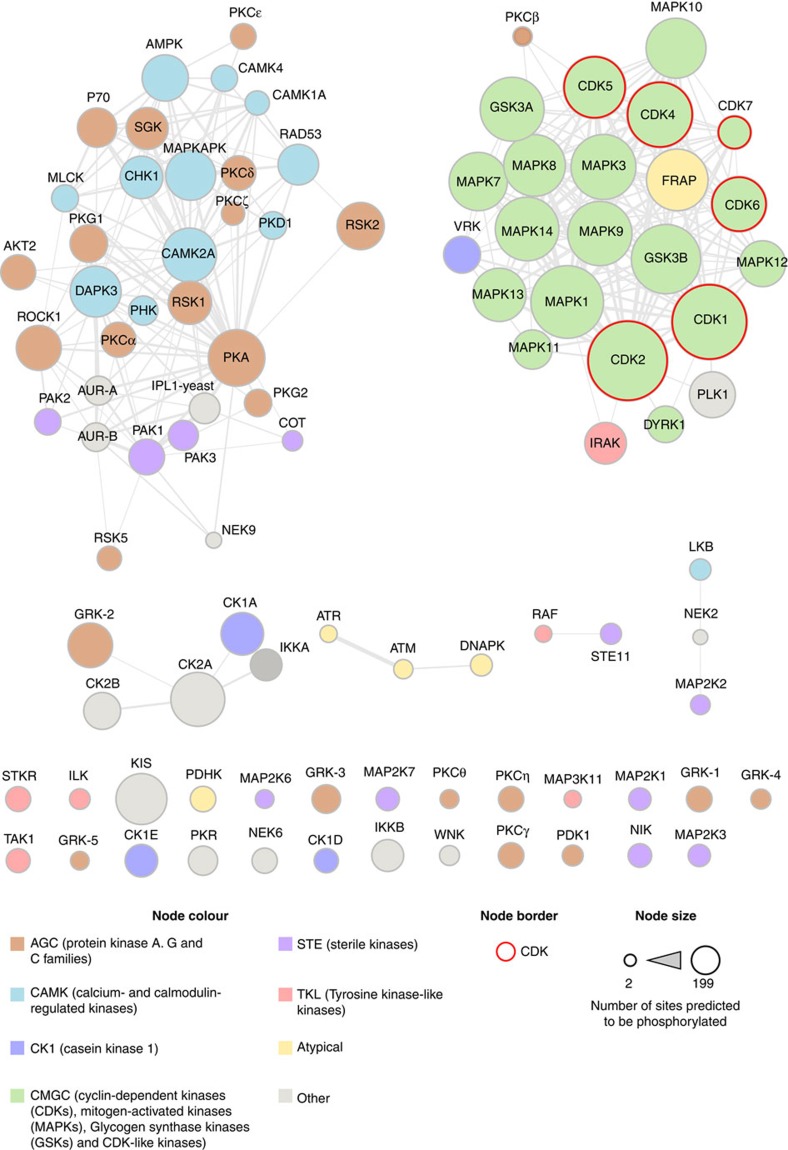
Serine/threonine kinases predicted to phosphorylate adhesion complex proteins. Phosphorylation sites identified by phosphoproteomic analysis of isolated adhesion complexes were searched using the kinase prediction tool GPS (version 2.1)^31^. All serine/threonine kinases predicted to phosphorylate the identified phosphorylation sites are displayed. Each node represents an individual kinase or group/family of kinases, and nodes are coloured according to kinase group (see key for details). An edge connecting two nodes indicates that the corresponding kinase groups were predicted to phosphorylate at least one common residue. Nodes are clustered according to connectivity (that is, clustered nodes were predicted to phosphorylate similar residues). Node size corresponds to the total number of adhesion complex phosphorylation sites that were predicted to be phosphorylated by the corresponding kinase. Kinases belonging to the CDK group are highlighted with a red border.

To identify protein kinases that are potentially involved in adhesion signalling, the GPS kinase prediction tool^31^ was used to interrogate adhesion complex phosphorylation sites (Fig. 5 and Supplementary Fig. 3). Of the 1,109 phosphorylation sites detected, 865 (78%) were identified as potential substrates for a particular protein kinase or group/family of protein kinases (Supplementary Data 9).

In addition to adhesion-associated kinases such as FAK and Src (Supplementary Fig. 3), the kinase prediction tool identified a large number of kinases that do not have well-characterized roles in integrin signalling (Fig. 5). For example, serine/threonine kinases of the CMGC group (including the CDK, glycogen synthase kinase and mitogen-activated protein kinase families) and the AGC group (including the protein kinase A and C families) were predicted to phosphorylate particularly large numbers of the phosphorylation sites identified from adhesion complex proteins (Fig. 5).

**Figure 6:**
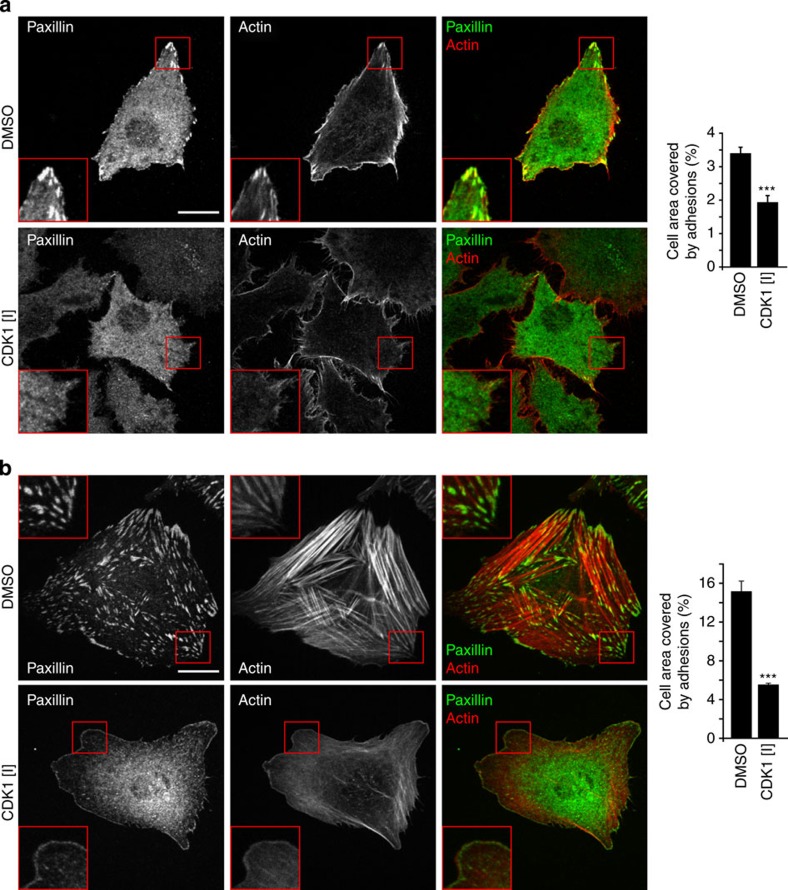
Effect of CDK1 inhibition on cell adhesion. A375 cells (**a**) and HFFs (**b**) were spread on FN for 60 min before treatment with the CDK1-specific inhibitor RO-3306 (CDK [I]) or DMSO for 60 min. Adhesion sites were visualized by immunofluorescence staining for paxillin (green) and the actin cytoskeleton was visualized by staining with Texas Red-conjugated phalloidin (red). For both cell types, the cell area covered by adhesion sites was quantified and is displayed to the right of immunofluorescence images (*n*=3, quantification based on 60 cells for each cell type). Scale bar, 20 μm. Error bars represent s.e.m. (***P<0.005; Student’s *t*-test).

CDKs play a fundamental role in regulating the cell cycle^32^, but are not generally considered to be involved in the regulation of cell adhesion or to phosphorylate substrates within adhesion complexes. However, the kinase prediction tool identified a large number of phosphorylation sites from adhesion complex proteins as potential CDK targets (CDK1, 185 sites; CDK2, 199 sites; CDK4, 156 sites; CDK5, 145 sites; CDK6, 126 sites; CDK7, 59 sites; Supplementary Data 9), suggesting that these kinases may play a role in adhesion signalling. Therefore, to validate the kinase prediction tool results, the impact of CDK1 inhibition on cell adhesion and spreading was tested. Treatment of A375 cells and human foreskin fibroblasts (HFFs) with the CDK1-specific inhibitor RO-3306 (refs 33, 34) triggered the disassembly of focal adhesions and a decrease in the area of cells covered by adhesions, leaving only small adhesions at the cell periphery that resembled nascent adhesions (Fig. 6a,b). Furthermore, treatment of cells with two alternative inhibitors of CDK1 activity also resulted in a significant decrease in the area of cells covered by adhesions (Supplementary Fig. 4). These findings highlight a role for CDK1 in the regulation of adhesion sites in non-dividing cells, and illustrate how the global analysis of adhesion site-specific phosphorylation events can be utilized to gain novel insights into the signalling networks that function downstream of adhesion receptor ligation.

## Discussion

The ligation of integrin receptors by their ECM ligands initiates highly complex intracellular signalling pathways that influence various fundamental aspects of cell behaviour. However, a comprehensive understanding of the molecular mechanisms that translate cell–ECM interactions into functional responses is lacking. To address this issue, we have generated the first global view of the protein and phosphoprotein composition of adhesion complexes. Although it has been previously postulated that phosphorylation plays a widespread role in regulating adhesion complex proteins^6^,^7^, this study provides the first experimental evidence of the high abundance of phosphorylation within adhesion complexes, and confirms the pivotal role played by this post-translational modification in adhesion signalling.

Many of the identified phosphorylation sites belonged to well-characterized adhesion complex proteins and represented established adhesion-induced signalling events. Notably, this coverage of adhesion-associated phosphoproteins was superior to that observed when more complex cell lysates were utilized (both here and in previous adhesion-based studies^11^,^22^). These findings support the notion that analysing subcellular compartments increases the sensitivity of detection for lower abundance phosphorylation sites^35^. However, notwithstanding the clear benefits achieved through specific analysis of adhesion complex proteins, it should be highlighted that this approach comes at the expense of detecting more distal signalling events within the cell that are induced by adhesion. Thus, in-depth analysis of the whole-cell phosphoproteome remains an important goal for a comprehensive analysis of adhesion receptor signalling.

The adhesion site-specific analysis performed here revealed a general enrichment of phosphotyrosine within adhesion complexes compared with whole-cell phosphoproteomes, an observation that is consistent with previous immunofluorescence analyses^18^,^19^,^20^,^21^. Indeed, it has been speculated that, in contrast to serine/threonine phosphorylation that existed before the evolution of integrin receptors in metazoa, tyrosine phosphorylation-based signalling mechanisms developed concomitantly with the emergence of integrins^36^. Thus, the onset of cell signalling through tyrosine kinases and phosphatases may have been a key enabling event in the evolution of multicellular organisms^36^,^37^. While the data collected here support this hypothesis, the high number of serine/threonine phosphorylation sites identified from adhesion complex proteins should not be overlooked. More than 90% of the phosphorylated residues identified from adhesion complex proteins in the present study were serines and threonines (82 and 11%, respectively). This suggests that serine/threonine phosphorylation events, relatively underappreciated in the context of cell adhesion until now, play a key role in adhesion signalling.

Validation of the phosphoproteomic data collected here revealed that all tested phosphorylation sites were present within adhesion complexes. However, while some phosphorylation sites were shown by immunoblotting to be dramatically upregulated by adhesion (for example, FAK pY397 and pY576 and paxillin pY118) others were shown by immunoblotting to be present at similar levels in the lysates of adherent and non-adherent cells (for example, FAK pY407, MYPT1 pT696 and cortactin pY421), raising questions as to the extent of adhesion-dependent upregulation of these residues. One explanation is that a small proportion of the total cellular pool of these proteins is phosphorylated on certain residues at adhesion sites, and a high background of the same phosphorylation site elsewhere in the cell prohibited the detection of its upregulation by immunoblotting using WCLs. Alternatively, it is possible that these phosphorylation sites are not upregulated in response to cell spreading on FN. Instead, some proteins may be recruited to adhesion sites in a prephosphorylated state, potentially maintaining preassembled protein complexes that have recently been shown to be recruited to adhesion sites from the cytosol^38^. Conversely, these residues could represent ‘passenger’ phosphorylation sites that are phosphorylated before their recruitment to adhesion sites, but do not perform a function within adhesion complexes. Elucidating which of these scenarios best explains the findings of this study requires further work. In any case, it is clear that two different types of phosphorylation site are found at adhesion complexes: one that is present at very low levels in non-adherent cells and undergoes substantial upregulation on cell spreading on FN, and a second that is present at high basal levels in non-adherent cells. It is notable that this insight was only made possible as a result of the spatial context afforded by analysing isolated adhesion complexes. The distinction between phosphorylated residues that are present within adhesion complexes versus those that are substantially upregulated in an adhesion-dependent manner would not have been achievable using cell lysates.

Using the phosphoproteomic data generated from isolated adhesion complexes, we have highlighted a large number of kinases that are potentially involved in regulating adhesion complex proteins. To confirm the validity of these findings, we have shown that CDK1 plays a role in regulating the formation of adhesion complexes. Although CDK activity is typically associated with regulating the cell cycle in the nucleus, previous studies have revealed a role for different members of the CDK family in regulating adhesion complex proteins including talin^39^,^40^, paxillin^41^, actopaxin^42^ and filamin-A^43^. Furthermore, increased expression of the integrin heterodimer αvβ3 has been shown to upregulate CDK activity and thereby promote cell migration^44^, and inhibition of CDK has previously been shown to block the adhesion and migration of leukocytes^45^. Therefore, the data collected here and elsewhere support the notion that CDK activity is not restricted to regulation of the cell cycle, but is also stimulated by ECM engagement and is involved in regulating cell adhesion and migration. It will be interesting to determine whether the many other putative novel adhesion-associated kinases identified in this study play a similarly important role in adhesion signalling.

In summary, the data presented here represent a catalogue of the phosphorylation events found specifically within adhesion complexes, and as such constitute a valuable resource to improve our understanding of the molecular mechanisms through which adhesion controls many aspects of cell behaviour.

## Methods

### Cell culture

A375-SM human melanoma cells (provided by I.J. Fidler, MD Anderson Cancer Center, Houston, TX, USA) and HFFs were maintained in Dulbecco’s modified Eagle’s medium (Sigma-Aldrich, Poole, UK) supplemented with 10% (v/v) fetal calf serum (Lonza Bioscience, Slough, UK) and 2 mM L-glutamine at 37 °C, 5% (v/v) CO_2_.

### Reagents

Monoclonal antibodies used were mouse anti-β1 integrin (JB1A; 1:1,000; provided by J.A. Wilkins, University of Manitoba, Winnipeg, MB, Canada), mouse anti-FAK (clone 77; 1:1,000; BD Biosciences, Oxford, UK; 610088), rabbit anti-FAK pY397 (clone 141-9; 1:1,000; Invitrogen, Paisley, UK; 44–625G), mouse anti-paxillin (clone 349; 1:10,000 for immunoblotting, 1:200 for immunofluorescence; BD Biosciences; 610051), mouse anti-HSP90 (EMD-17D7; 1:1,000; Millipore, Billerica, MA, USA; CA1023-50UG), rabbit anti-lamin B1 (D4Q4Z, 1:1,000; Cell Signaling Technology, Danvers, MA, USA; 12586S), mouse anti-mitochondrial HSP70 (JG1; 1:1,000; Thermo Fisher Scientific, Waltham, MA, USA; MA3028), rabbit anti-SHP2 pY580 (D66F10; 1:1,000; Cell Signaling Technology; 5431S), mouse anti-talin (8D4; 1:1,000; Sigma-Aldrich; T3287) and mouse anti-TfR (H68.4; 1:1,000; Invitrogen; 13–6890). Polyclonal antibodies used were rabbit anti-α5 integrin (H-104, 1:1,000; Santa Cruz Biotechnology, Santa Cruz, CA, USA; sc-10729), rabbit anti-calnexin (1:1,000; Enzo Life Sciences, Exeter, UK; ADI-SPA-860), rabbit anti-cortactin pY421 (1:1,000 for immunoblotting, 1:100 for immunofluorescence; Cell Signaling Technology; 4569S), rabbit anti-FAK pY407 (1:1,000 for immunoblotting, 1:100 for immunofluorescence; Invitrogen; 44–650G), rabbit anti-FAK pY576 (1:1,000; Invitrogen; 44–652G), rabbit anti-MYPT1 pT696 (1:1,000; Millipore; ABS45), rabbit anti-PKCδ pY313 (1:1,000 for immunoblotting, 1:100 for immunofluorescence; Cell Signaling Technology; 2055S), rabbit anti-paxillin pY118 (1:1,000; Invitrogen; 44–722G) and rabbit anti-Src pY416 (1:1,000; Cell Signaling Technology; 2101S). Secondary Alexa-Fluor 680-conjugated (1:5,000; Invitrogen) or DyLight 800-conjugated (1:5,000; Cell Signaling Technology) antibodies were used for immunoblotting. Secondary Alexa-Fluor 647-conjugated anti-mouse (1:200) and Alexa-Fluor 488-conjugated anti-rabbit (1:200) antibodies were used for immunofluorescence (both from Invitrogen).

### Generation of isolated adhesion complexes and cell lysates

Subconfluent A375-SM cells in culture were detached with trypsin (Sigma-Aldrich) and resuspended in Dulbecco’s modified Eagle’s medium supplemented with 25 mM HEPES (Sigma-Aldrich). Cells were then incubated in suspension for 20 min at 37 °C to downregulate ECM adhesion signalling events. Cells were then allowed to spread on 55-cm^2^ dishes (Corning, NY, USA) coated with 10 μg ml^−1^ FN (Sigma-Aldrich) or 10 μg ml^−1^ Tf (Sigma-Aldrich) for 120 min at 37 °C, 8% (v/v) CO_2_.

To isolate adhesion complexes, cells spread on FN or Tf were incubated with the membrane permeable crosslinker dimethyl-3, 3′-dithiobispropionimidate (DTBP, Sigma-Aldrich; 3 mM, 30 min). Cells were then washed twice with PBS (Sigma-Aldrich) and DTBP was quenched using 1 M Tris (pH 8, 10 min), after which the cells were again washed twice using PBS and incubated in PBS at 4 °C. Cell bodies were then removed by a combination of cell lysis in RIPA buffer (50 mM Tris-HCl, pH 8.0, 5 mM EDTA, 150 mM NaCl, 1% (w/v) Triton-X-100, 1% (w/v) sodium deoxycholate, 0.5% (w/v) SDS; 3 min) and a high-pressure water wash (10 s). Protein complexes left bound to FN or Tf were washed five times using PBS, recovered by scraping in 100 μl recovery solution (125 mM Tris-HCl, pH 6.8, 1% (w/v) SDS, 15% (v/v) β-mercaptoethanol) and incubated at 70 °C for 5–10 min. Where applicable, a proportion of each sample was precipitated from solution by addition of four volumes −20 °C acetone, incubated for 3.5–12 h on dry ice and resuspended in reducing sample buffer for SDS-polyacrylamide gel electrophoresis (SDS–PAGE) and immunoblot analyses.

To generate WCLs of cells spread on FN or Tf, the medium from each dish was aspirated and centrifuged (300 *g*, 4 min) to collect non-adherent cells. Lysates of adherent cells were then collected by scraping in lysis buffer (150 mM NaCl, 25 mM Tris, 2% (w/v) Triton-X-100, 0.5 mM AEBSF, 10 μg ml^−1^ leupeptin, 10 μg ml^−1^ aprotonin, 10 mM NaF, 2 mM Na_3_VO_4_, pH 7.4; 2 min). To ensure equal numbers of cells were used in all conditions, lysed cells were aspirated from each dish and mixed with the cell pellet formed by centrifugation of non-adherent cells collected from corresponding dishes. Finally, the non-solubilized material was removed by centrifugation (22,000 *g*, 15 min, 4 °C).

### Immunoblotting

Protein samples were separated by SDS–PAGE (4–12% Bis–Tris gels; Invitrogen) and transferred to nitrocellulose membrane (Whatman, Maidstone, UK). Membranes were blocked for 60 min at room temperature (RT) using Odyssey blocking buffer (Sigma-Aldrich), and then probed overnight with primary antibodies diluted in blocking buffer in Tris-buffered saline (TBS; 10 mM Tris-HCl, pH 7.4, 150 mM NaCl) containing 0.05% Tween-20 (TBST) at 4 °C. Membranes were washed for 30 min using PBS, and then incubated with the appropriate fluorophore-conjugated secondary antibody diluted in blocking buffer in TBST for 30 min at RT in the dark. Membranes were washed for 30 min in the dark using PBS, and then scanned using the Odyssey infrared imaging system (LI-COR Biosciences). Uncropped images of all membranes are displayed in Supplementary Figs 5 and 6.

### Proteolytic digestion and phosphopeptide enrichment

For proteomic analyses, protein samples were separated by SDS–PAGE. Samples were allowed to migrate approximately half way down a 4–12% Bis–Tris gel), stained for 60 min with Instant Blue (Expedeon, Cambridgeshire, UK), and washed in water overnight at 4 °C. Gel lanes were excised and divided into 15 slices, and each gel slice was then processed using an in-gel tryptic digestion procedure as described previously^46^ with modifications^8^.

For phosphoproteomic analyses, protein samples were reduced using 10 mM dithiotheitol for 60 min at 56 °C and then alkylated using 15 mM iodoacetamide (IAM; 45 min, RT). Proteins were precipitated from solution by addition of four volumes −20 °C acetone, incubated for 3.5–12 h on dry ice. Following centrifugation (16,000 *g*, 15 min, 4 °C), the precipitated protein pellet was washed twice with −20 °C acetone, and then dried for 20–30 min in a fume hood. Protein pellets were resuspended in 50 mM NH_4_HCO_3_ containing 0.2% (w/v) RapiGest Surfactant (Waters, Manchester, UK) and digested with trypsin (Promega, Madison, WI, USA) at an enzyme:substrate ratio of 1:50–1:100 (overnight, 37 °C). Digested samples were acidified to 0.5–1% (v/v) trifluoroacetic acid (TFA), incubated at 37 °C for 45 min, and centrifuged at 22,000 *g* for 10 min to remove water-insoluble RapiGest by-products.

Peptides were desalted using Oasis HLB sample extraction columns (Waters), and eluted in phosphopeptide enrichment binding solution (65% acetonitrile (ACN), 2% TFA, saturated with glutamic acid, pH 2–3). Phosphopeptides were enriched by incubation with titanium dioxide enrichment beads (Glygen, Columbia, MD, USA) for 60 min at RT. Beads were then washed three times, firstly using binding solution, then wash solution 1 (0.5% (v/v) TFA, 65% (v/v) ACN) and finally wash solution 2 (65% (v/v) ACN, 0.1% (v/v) TFA). Phosphopeptides were acidified to pH 2–3 using TFA, and then eluted from beads by incubating with elution solution (300 mM ammonium hydroxide, 5% (v/v) ACN; 5 min, RT). Phosphopeptide-enriched samples were desalted twice on reverse-phase ZipTips containing C_18_ media (Millipore). Peptides were eluted using 50% (v/v) ACN in 0.1% (v/v) formic acid (FA), dried to completion and resuspended in 5% ACN, 0.1% FA for tandem mass spectrometric (LC-MS/MS) analysis.

### LC-MS/MS analyses and data analysis

Peptide analysis by LC-MS/MS was performed using an UltiMate 3000 Rapid Separation LC (RSLC, Dionex Corporation, Sunnyvale, CA, USA) coupled to an Orbitrap Elite mass spectrometer (Thermo Fisher Scientific). Peptides were separated on a bridged ethyl hybrid C_18_ analytical column (250 mm × 75 μm i.d., 1.7 μm particle size, Waters) over a 45-min (proteomic analyses) or 165 min (phosphoproteomic analyses) gradient from 8 to 33% (v/v) ACN in 0.1% (v/v) FA. LC-MS/MS analyses were operated in data-dependent mode to automatically select peptides for fragmentation by collision-induced dissociation. For phosphoproteomic analyses, multistage activation was enabled to fragment product ions resulting from neutral loss of phosphoric acid, as described previously^47^.

Tandem mass spectra were extracted using extract_msn (Thermo Fisher Scientific) executed in Mascot Daemon (version 2.2.2; Matrix Science). Peak lists were searched against a modified version of the IPI Human database (version 3.70), modified to contain additional contaminant and reagent sequences of non-human origin. Database searching was performed using an in-house Mascot server (version 2.2.03; Matrix Science)^48^. Only tryptic peptides were considered. A maximum of one missed cleavage was permitted, with a peptide mass tolerance of 5 p.p.m. and an MS/MS tolerance of 0.5 Da. Monoisotopic precursor mass values were used, and only doubly and triply charged precursor ions were considered. Carbamidomethylated cysteine was set as a fixed modification, while serine, threonine and tyrosine phosphorylation and methionine oxidation were set as variable modifications. Data generated by Mascot were validated using Scaffold (version 3.6.5, Proteome Software, Portland, OR, USA). Protein identifications were accepted if they were assigned at least two unique peptides and could be established with at least 90% probability at the peptide level and at least 99% probability at the protein level. Individual phosphopeptide identifications (based on one unique peptide) were accepted if they could be established with at least 95% probability at the peptide level and at least 80% probability at the protein level. All detected proteins and phosphopeptides are displayed in Supplementary Data 1 and 3, respectively.

To identify adhesion complex-specific proteins and phosphopeptides, adhesion complexes and controls were compared by spectral counting. For protein identification, all proteins exhibiting at least a twofold enrichment to adhesion complexes compared with control identifications were deemed adhesion complex-specific (based on mean normalized spectral counts across three biological replicates; minimum of six spectral counts per protein; Supplementary Data 2). For phosphopeptide identification, all phosphopeptides exhibiting at least a ninefold enrichment to adhesion complexes compared with control identifications (>2 s.d. above mean fold change) were deemed adhesion complex-specific (based on mean spectral counts across three adhesion complex isolations (five LC-MS/MS analyses); minimum of 2 spectral counts per peptide; Supplementary Data 4). Phosphorylation site localization scores were assigned according to the Mascot Delta score^49^ using an in-house set-up.

### GO enrichment analyses

GO enrichment analyses were performed using the online bioinformatic tools available via the Database for Annotation, Visualization and Integrated Discovery (DAVID; version 6.7; http://david.abcc.ncifcrf.gov/home.jsp)^50^,^51^, utilizing two of the available GO categories: molecular function and BP. Protein lists were searched using the IPI IDs, as assigned by Scaffold or as supplied for published data sets^23^,^24^. The background data set used for analyses was the *Homo sapiens* genome. GO terms with Bonferroni-corrected *P* value <0.05, and at least two proteins per term were considered significantly enriched.

### PPI network analyses

PPI network analyses were performed using Cytoscape (version 2.8.1)^52^. Proteins were mapped onto a merged human interactome comprising PPIs listed in the Protein Interaction Network Analysis (release date 10th December 2012)^53^ and a literature-curated database of integrin-associated adhesion complex proteins^7^.

### Kinase prediction analysis

Adhesion complex-specific phosphorylation sites were analysed using the Group-based Prediction System (GPS; version 2.1.2)^31^. To minimize false positives, the high threshold false positive rate was applied (2% for serine/threonine kinases; 4% for tyrosine kinases). Of the 31 phosphorylation sites in the data set that have previously been identified as a substrate for a particular kinase, 22 (70%) were matched to the correct kinase by the kinase prediction tool (Supplementary Data 10). These included sites such as pY576 and pY577 on FAK that are well-defined substrates for the tyrosine kinase Src, as well as the autophosphorylation site on FAK pY397. Of the remaining nine phosphorylation sites that were not matched to the correct kinase, two were identified as substrates for kinases that are not components of the kinase prediction tool, four were predicted to be phosphorylated by a different isoform or family member of the reported upstream kinase, and four were not assigned a predicted upstream kinase.

### Immunofluorescence microscopy

A375-SM cells or HFFs were spread for 120 min at 37 °C, 5% (v/v) CO_2_ on glass-bottomed dishes (10 mm glass diameter, 35 mm plate diameter; MatTek Co., Ashland, MA, USA) coated with 10 μg ml^−1^ FN. Where applicable, spread cells were incubated with the CDK1-specific inhibitor RO-3306 (10 μM; Millipore), CGP74514A (5 μM, Millipore), Roscovitone (20 μM, Cell Signaling) or DMSO for 60 min at 37 °C, 8% (v/v) CO_2_. Cells were fixed in 4% (w/v) paraformaldehyde for 10 min, washed twice with PBS and permeabilized using 0.5% (w/v) Triton-X-100 in PBS for 5 min. Cells were then washed twice with PBS and glass-bottomed dishes were blocked using 0.1 M glycine in PBS (60 min, RT). Cells were incubated with primary antibodies (30 min, RT), and then washed with PBS and incubated for 30 min with the appropriate secondary antibodies and, where applicable, Alexa 594-conjugated Phalloidin (Invitrogen). Finally, glass-bottomed dishes were washed with PBS a further three times before imaging.

Images were acquired on an inverted confocal microscope (TCS SP5 Acousto-Optical Beam Splitter; Leica, Wetzlar, Germany) using a × 63 objective (HCX Plan Apochromat, NA 1.25) and LCS software (Leica). Alternatively, images were acquired on a spinning-disk confocal inverted microscope (Marianas; 3i, Denver, CO, USA) using a × 63 objective (Plan Apochromat, NA 1.4) and SlideBook 5.0 software (3i).

## Additional information

**Accession codes:** The mass spectrometry proteomics data have been deposited to the ProteomeXchange Consortium^54^ via the PRIDE partner repository with the data set identifier PXD001578 and DOI 10.6019/PXD001578.

**How to cite this article:** Robertson, J. *et al*. Defining the phospho-adhesome through the phosphoproteomic analysis of integrin signalling. *Nat. Commun.* 6:6265 doi: 10.1038/ncomms7265 (2015).

## Supplementary Material

Supplementary Figures Supplementary Figures 1-6.

Supplementary Data 1 Proteins identified from isolated complexes. Three biological replicate proteomic analyses (A, B and C) of complexes isolated from cells spread on FN or Tf were performed. The table shows all proteins identified following database searches using MASCOT and data validation using Scaffold. For each protein, the number of spectra identified from each replicate is shown, along with the fold enrichment to FN based on mean normalised spectral counts (normalised to total number of IDs and molecular weight).

Supplementary Data 2 Adhesion complex-specific proteins. Three biological replicate proteomic analyses (A, B and C) of complexes isolated from cells spread on FN or Tf were performed. The table shows all proteins that were deemed adhesion complex-specific following a quantitative comparison between adhesion complexes and controls (see methods). For each protein, the number of spectra identified from each replicate is shown, along with the fold enrichment to FN based on mean normalised spectral counts (normalised to total number of IDs and molecular weight).

Supplementary Data 3 Phosphorylation sites identified from isolated complexes. Three separate analyses (A, B and C) of complexes isolated from cells spread on FN or Tf were performed (two using approximately 100 μg starting material and one using approximately 400 μg starting material). The table shows all phosphorylation sites identified following database searches using MASCOT and data validation using Scaffold. For each phosphorylation site, the number of spectra identified from replicate LC-MS/MS analyses of phosphopeptide-enriched samples is shown (with the exception of analysis B, in which only one LC-MS/MS analysis was performed).

Supplementary Data 4 Adhesion complex-specific phosphorylation sites. Three separate analyses (A, B and C) of complexes isolated from cells spread on FN or Tf were performed (two using approximately 100 μg starting material and one using approximately 400 μg starting material). The table shows all phosphorylation sites that were deemed adhesion complex-specific following a quantitative comparison between adhesion complexes and controls (see methods). For each phosphorylation site, the number of spectra identified from replicate LC-MS/MS analyses of phosphopeptide-enriched samples is shown (with the exception of analysis B, in which only one LC-MS/MS analysis was performed). An intra peptide score (indicating confidence in correct localisation of the phosphorylation site on the peptide; scores lower than 20 are ambiguous) and an inter peptide score (indicating confidence in correct identification of the peptide; scores lower than 20 highlight poor confidence assignments) are also displayed (in both cases, a score of -1 indicates an infinite score, i.e. very high confidence). Additionally, Uniprot was used to identify whether the identified phosphorylation sites have been reported elsewhere, or whether they have previously been mutated (along with any reported effect of the mutation). Uniprot was also used to identify whether the identified sites are within known domains, regions, binding sites or motifs of the protein. N/D, not determined.

Supplementary Data 5 Gene Ontology analysis of proteins identified by phosphoproteomic analysis of isolated adhesion complexes. All 499 proteins identified by phosphoproteomic analysis of isolated adhesion complexes were subjected to functional enrichment analysis using the biological process ontology category. The number of proteins (count), enrichment values and -log10 corrected P-values for all significantly enriched (P<0.05) terms are displayed.

Supplementary Data 6 Gene Ontology analysis of proteins identified by phosphoproteomic analysis of the WCLs of A375 cells. The 735 proteins identified by phosphoproteomic analysis of the WCLs of A375 cells adherent to FN were subjected to functional enrichment analysis using the biological process ontology category. The number of proteins (count), enrichment values and -log10 corrected P-values for all significantly enriched (P<0.05) terms are displayed.

Supplementary Data 7 Gene ontology analysis of proteins identified by phosphoproteomic analysis of the WCLs of HeLa cells. The 5192 proteins identified by phosphoproteomic analysis of the WCLs of HeLa cells23 were subjected to functional enrichment analysis using the biological process ontology category. The number of proteins (count), enrichment values, and -log10 corrected P-values for all significantly enriched (P<0.05) terms are displayed.

Supplementary Data 8 Gene ontology analysis of proteins identified by phosphoproteomic analysis of the WCLs of hESCs. The 4061 proteins identified by phosphoproteomic analysis of the WCLs of human embryonic stem cell (hESCs)24 were subjected to functional enrichment analysis using the biological process ontology category. The number of proteins (count), enrichment values, and -log10 corrected P-values for all significantly enriched (P<0.05) terms are displayed.

Supplementary Data 9 Kinases predicted to phosphorylate adhesion complex proteins. Phosphorylation sites identified by phosphoproteomic analysis of isolated adhesion complexes were searched using the kinase prediction tool GPS (version 2.1). The table lists all phosphorylation sites that were assigned a predicted kinase (863 in total). For each phosphorylation site, the predicted kinase or group/family of kinases is displayed, along with the kinase score (reflecting the confidence of the assigned kinase) and the kinase score cut off (set to the most stringent value, giving FDRs of 2% for serine/threonine kinases and 4% for tyrosine kinases).

Supplementary Data 10 Phosphorylation sites identified from isolated adhesion complexes that are known substrates for protein kinases. All 31 phosphorylation sites that have previously been reported as substrates for a specific protein kinase (as listed in Uniprot) are displayed. The final column provides information on whether the known upstream kinase was correctly identified as a potential upstream kinase for each phosphorylation site.

## References

[b1] Legate K. R., Wickström S. A. & Fässler R. Genetic and cell biological analysis of integrin outside-in signaling. Genes Dev. 23, 397–418 (2009). 19240129 10.1101/gad.1758709

[b2] Schwartz M. A. Integrin signaling revisited. Trends Cell Biol. 11, 466–470 (2001). 11719050 10.1016/s0962-8924(01)02152-3

[b3] Wolfenson H., Lavelin I. & Geiger B. Dynamic regulation of the structure and functions of integrin adhesions. Dev. Cell 24, 447–458 (2013). 23484852 10.1016/j.devcel.2013.02.012 PMC3878073

[b4] Wehrle-Haller B. & Imhof B. A. Integrin-dependent pathologies. J. Pathol. 200, 481–487 (2003). 12845615 10.1002/path.1399

[b5] Winograd-Katz S. E., Fässler R., Geiger B. & Legate K. R. The integrin adhesome: from genes and proteins to human disease. Nat. Rev. Mol. Cell Biol. 15, 273–288 (2014). 24651544 10.1038/nrm3769

[b6] Zaidel-Bar R. & Geiger B. The switchable integrin adhesome. J. Cell Sci. 123, 1385–1388 (2010). 20410370 10.1242/jcs.066183 PMC2858016

[b7] Zaidel-Bar R., Itzkovitz S., Ma’ayan A., Iyengar R. & Geiger B. Functional atlas of the integrin adhesome. Nat. Cell Biol. 9, 858–867 (2007). 17671451 10.1038/ncb0807-858 PMC2735470

[b8] Humphries J. D. . Proteomic analysis of integrin-associated complexes identifies RCC2 as a dual regulator of Rac1 and Arf6. Sci. Signal. 2, ra51 (2009). 19738201 10.1126/scisignal.2000396 PMC2857963

[b9] Kuo J.-C., Han X., Hsiao C.-T., Yates Iii J. R. & Waterman C. M. Analysis of the myosin-II-responsive focal adhesion proteome reveals a role for β-Pix in negative regulation of focal adhesion maturation. Nat. Cell Biol. 13, 383–393 (2011). 21423176 10.1038/ncb2216 PMC3279191

[b10] Schiller H. B., Friedel C. C., Boulegue C. & Fassler R. Quantitative proteomics of the integrin adhesome show a myosin II-dependent recruitment of LIM domain proteins. EMBO Rep. 12, 259–266 (2011). 21311561 10.1038/embor.2011.5 PMC3059911

[b11] Schiller H. B. . β1- and αv-class integrins cooperate to regulate myosin II during rigidity sensing of fibronectin-based microenvironments. Nat. Cell Biol. 15, 625–636 (2013). 23708002 10.1038/ncb2747

[b12] Byron A., Humphries J. D., Craig S. E., Knight D. & Humphries M. J. Proteomic analysis of α4β1 integrin adhesion complexes reveals α-subunit-dependent protein recruitment. Proteomics 12, 2107–2114 (2012). 22623428 10.1002/pmic.201100487 PMC3472074

[b13] Geiger T. & Zaidel-Bar R. Opening the floodgates: proteomics and the integrin adhesome. Curr. Opin. Cell Biol. 24, 562–568 (2012). 22728062 10.1016/j.ceb.2012.05.004

[b14] Hunter T. Why nature chose phosphate to modify proteins. Philos. Trans. R. Soc. B Biol. Sci. 367, 2513–2516 (2012). 10.1098/rstb.2012.0013 PMC3415839 22889903

[b15] Mitra S. K. & Schlaepfer D. D. Integrin-regulated FAK–Src signaling in normal and cancer cells. Curr. Opin. Cell Biol. 18, 516–523 (2006). 16919435 10.1016/j.ceb.2006.08.011

[b16] Defilippi P., Di Stefano P. & Cabodi S. p130Cas: a versatile scaffold in signaling networks. Trends Cell Biol. 16, 257–263 (2006). 16581250 10.1016/j.tcb.2006.03.003

[b17] Deakin N. O. & Turner C. E. Paxillin comes of age. J. Cell Sci. 121, 2435–2444 (2008). 18650496 10.1242/jcs.018044 PMC2522309

[b18] Panetti T. S. Tyrosine phosphorylation of paxillin, FAK, and p130CAS: effects on cell spreading and migration. Front. Biosci. 7, d143–d150 (2002). 11779709 10.2741/A771

[b19] Kirchner J., Kam Z., Tzur G., Bershadsky A. D. & Geiger B. Live-cell monitoring of tyrosine phosphorylation in focal adhesions following microtubule disruption. J. Cell Sci. 116, 975–986 (2003). 12584242 10.1242/jcs.00284

[b20] Iyer V. V., Ballestrem C., Kirchner J., Geiger B. & Schaller M. D. Measurement of protein tyrosine phosphorylation in cell adhesion. Methods Mol. Biol. 294, 289–302 (2005). 15576919 10.1385/1-59259-860-9:289

[b21] Zaidel-Bar R., Ballestrem C., Kam Z. & Geiger B. Early molecular events in the assembly of matrix adhesions at the leading edge of migrating cells. J. Cell Sci. 116, 4605–4613 (2003). 14576354 10.1242/jcs.00792

[b22] Chen Y. . Combined integrin phosphoproteomic analyses and siRNA-based functional screening identified key regulators for cancer cell adhesion and migration. Cancer Res. 69, 3713–3720 (2009). 19351860 10.1158/0008-5472.CAN-08-2515 PMC2669841

[b23] Olsen J. V. . Quantitative phosphoproteomics reveals widespread full phosphorylation site occupancy during mitosis. Sci. Signal. 3, ra3–ra3 (2010). 20068231 10.1126/scisignal.2000475

[b24] Rigbolt K. T. G. . System-wide temporal characterization of the proteome and phosphoproteome of human embryonic stem cell differentiation. Sci. Signal. 4, rs3 (2011). 21406692 10.1126/scisignal.2001570

[b25] Schaller M. D. . Autophosphorylation of the focal adhesion kinase, pp125FAK, directs SH2-dependent binding of pp60src. Mol. Cell. Biol. 14, 1680–1688 (1994). 7509446 10.1128/mcb.14.3.1680-1688.1994 PMC358526

[b26] Nojima Y. . Integrin-mediated cell adhesion promotes tyrosine phosphorylation of p130Cas, a Src homology 3-containing molecule having multiple Src homology 2-binding motifs. J. Biol. Chem. 270, 15398–15402 (1995). 7541040 10.1074/jbc.270.25.15398

[b27] Schaller M. D. & Parsons J. T. pp125FAK-dependent tyrosine phosphorylation of paxillin creates a high-affinity binding site for Crk. Mol. Cell. Biol. 15, 2635–2645 (1995). 7537852 10.1128/mcb.15.5.2635 PMC230493

[b28] Kanshin E., Michnick S. & Thibault P. Sample preparation and analytical strategies for large-scale phosphoproteomics experiments. Semin. Cell Dev. Biol. 23, 843–853 (2012). 22683502 10.1016/j.semcdb.2012.05.005

[b29] Olsen J. V. . Global, *in vivo*, and site-specific phosphorylation dynamics in signaling networks. Cell 127, 635–648 (2006). 17081983 10.1016/j.cell.2006.09.026

[b30] Rosales J. L. & Isseroff R. R. Increased expression of a high molecular weight (130 KD) protein kinase C isoform in a differentiation-defective ras-transfected keratinocyte line. J. Cell Physiol. 164, 509–521 (1995). 7544354 10.1002/jcp.1041640309

[b31] Xue Y. . GPS 2.0, a tool to predict kinase-specific phosphorylation sites in hierarchy. Mol. Cell Proteomics 7, 1598–1608 (2008). 18463090 10.1074/mcp.M700574-MCP200 PMC2528073

[b32] Malumbres M. & Barbacid M. Mammalian cyclin-dependent kinases. Trends Biochem. Sci. 30, 630–641 (2005). 16236519 10.1016/j.tibs.2005.09.005

[b33] Vassilev L. T. . Selective small-molecule inhibitor reveals critical mitotic functions of human CDK1. Proc. Natl Acad. Sci. USA 103, 10660–10665 (2006). 16818887 10.1073/pnas.0600447103 PMC1502288

[b34] Vassilev L. T. Cell cycle synchronization at the G2/M phase border by reversible inhibition of CDK1. Cell Cycle 5, 2555–2556 (2006). 17172841 10.4161/cc.5.22.3463

[b35] Trost M., Bridon G., Desjardins M. & Thibault P. Subcellular phosphoproteomics. Mass Spectrom. Rev. 29, 962–990 (2010). 20931658 10.1002/mas.20297

[b36] Zaidel-Bar R. Evolution of complexity in the integrin adhesome. J. Cell Biol. 186, 317–321 (2009). 19667126 10.1083/jcb.200811067 PMC2728394

[b37] Lim W. A. & Pawson T. Phosphotyrosine signaling: evolving a new cellular communication system. Cell 142, 661–667 (2010). 20813250 10.1016/j.cell.2010.08.023 PMC2950826

[b38] Hoffmann J.-E., Fermin Y., Stricker R. L., Ickstadt K. & Zamir E. Symmetric exchange of multi-protein building blocks between stationary focal adhesions and the cytosol. eLife 3, (2014). 10.7554/eLife.02257 PMC4040925 24894463

[b39] Jin J.-K. . Talin1 phosphorylation activates β1 integrins: a novel mechanism to promote prostate cancer bone metastasis. Oncogene (2014). 10.1038/onc.2014.116 PMC4221586 24793790

[b40] Huang C. . Talin phosphorylation by Cdk5 regulates Smurf1-mediated talin head ubiquitylation and cell migration. Nat. Cell Biol. 11, 624–630 (2009). 19363486 10.1038/ncb1868 PMC2714540

[b41] Miyamoto Y. . Cdk5 regulates differentiation of oligodendrocyte precursor cells through the direct phosphorylation of paxillin. J. Cell Sci. 120, 4355–4366 (2007). 18042622 10.1242/jcs.018218

[b42] Curtis M., Nikolopoulos S. N. & Turner C. E. Actopaxin is phosphorylated during mitosis and is a substrate for cyclin B1/cdc2 kinase. Biochem. J. 363, 233–242 (2002). 11931650 10.1042/0264-6021:3630233 PMC1222471

[b43] Zhong Z. . Cyclin D1/cyclin-dependent kinase 4 interacts with filamin A and affects the migration and invasion potential of breast cancer cells. Cancer Res. 70, 2105–2114 (2010). 20179208 10.1158/0008-5472.CAN-08-1108 PMC2917898

[b44] Manes T. . Alpha(v)beta3 integrin expression up-regulates cdc2, which modulates cell migration. J. Cell Biol. 161, 817–826 (2003). 12771130 10.1083/jcb.200212172 PMC2199360

[b45] Liu L. . Cyclin-dependent kinase inhibitors block leukocyte adhesion and migration. J. Immunol. 180, 1808–1817 (2008). 18209078 10.4049/jimmunol.180.3.1808 PMC4623328

[b46] Shevchenko A. . A strategy for identifying gel-separated proteins in sequence databases by MS alone. Biochem. Soc. Trans. 24, 893–896 (1996). 8878870 10.1042/bst0240893

[b47] Schroeder M. J., Shabanowitz J., Schwartz J. C., Hunt D. F. & Coon J. J. A neutral loss activation method for improved phosphopeptide sequence analysis by quadrupole ion trap mass spectrometry. Anal. Chem. 76, 3590–3598 (2004). 15228329 10.1021/ac0497104

[b48] Perkins D. N., Pappin D. J., Creasy D. M. & Cottrell J. S. Probability-based protein identification by searching sequence databases using mass spectrometry data. Electrophoresis 20, 3551–3567 (1999). 10612281 10.1002/(SICI)1522-2683(19991201)20:18<3551::AID-ELPS3551>3.0.CO;2-2

[b49] Savitski M. M. . Confident phosphorylation site localization using the Mascot delta score. Mol. Cell Proteomics 10, (2011). 10.1074/mcp.M110.003830 PMC3033680 21057138

[b50] Dennis G. Jr . DAVID: Database for Annotation, Visualization, and Integrated Discovery. Genome Biol. 4, P3 (2003). 12734009 PMC193660

[b51] Huang D. W., Sherman B. T. & Lempicki R. A. Systematic and integrative analysis of large gene lists using DAVID bioinformatics resources. Nat. Protoc. 4, 44–57 (2009). 19131956 10.1038/nprot.2008.211

[b52] Smoot M. E., Ono K., Ruscheinski J., Wang P.-L. & Ideker T. Cytoscape 2.8: new features for data integration and network visualization. Bioinformatics 27, 431–432 (2011). 21149340 10.1093/bioinformatics/btq675 PMC3031041

[b53] Wu J. . Integrated network analysis platform for protein-protein interactions. Nat. Methods 6, 75–77 (2009). 19079255 10.1038/nmeth.1282

[b54] Vizcaíno J. A. . ProteomeXchange provides globally coordinated proteomics data submission and dissemination. Nat. Biotechnol. 32, 223–226 (2014). 24727771 10.1038/nbt.2839 PMC3986813

