# Supplementary material for: Defining the phospho-adhesome through the phosphoproteomic analysis of integrin signalling
Source: Nat Commun. 2015 Feb 13;6:6265. doi: 10.1038/ncomms7265 (PMC4338609; doi:10.1038/ncomms7265)
Supplement: Supplementary Figures — 1-6. [file ncomms7265-s1.pdf]

a

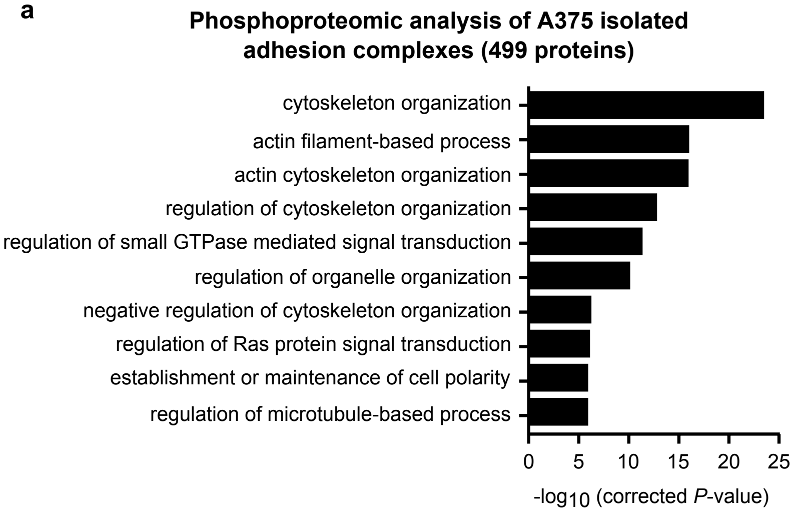

b

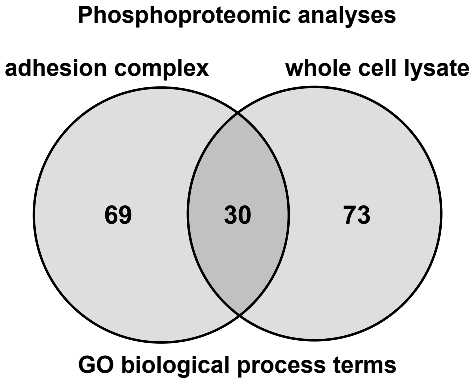

c

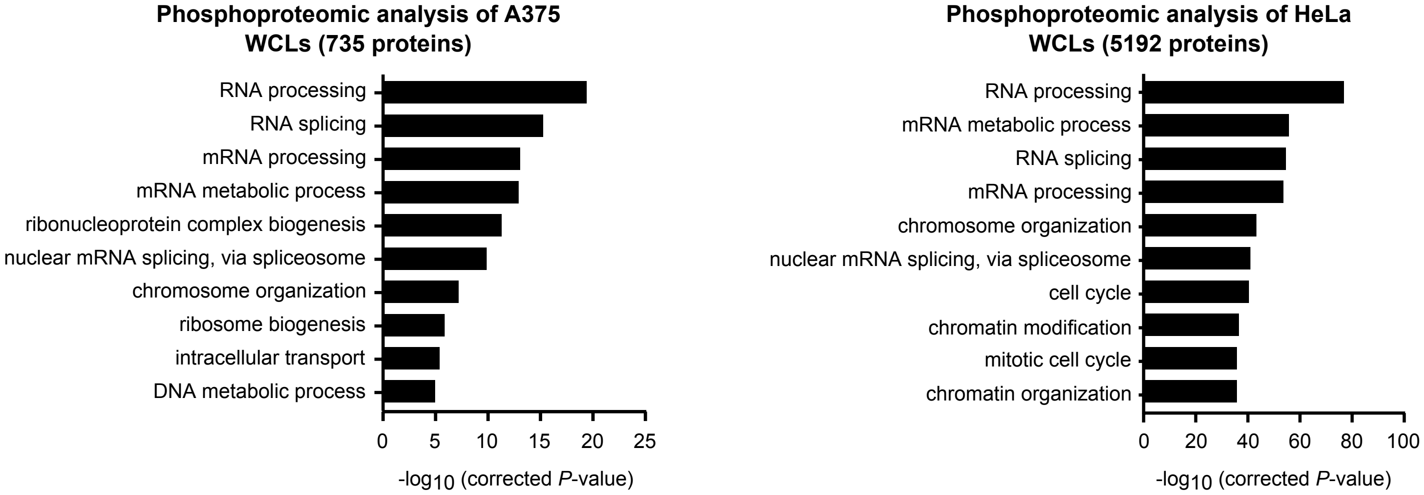

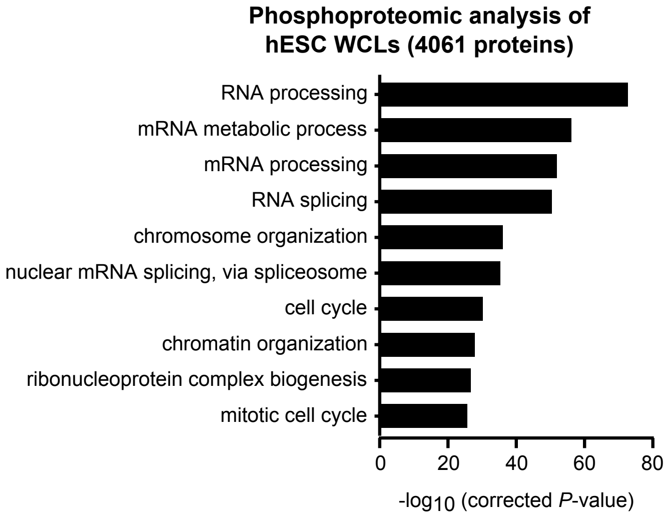

Supplementary Figure 1 Gene Ontology analysis of phosphoproteins identified from isolated adhesion complexes and whole cell lysates. (**a**) All 499 phosphoproteins identified by phosphoproteomic analysis of isolated adhesion complexes were searched for enrichment of biological process ontology terms. The -log10-transformed *P*-values for the ten highest ranking significantly enriched terms are displayed. (**b**) For comparison, a phosphoprotein dataset collected using the whole cell lysates (WCLs) of FN-spread A375 cells (735 proteins) was subjected to the same enrichment analysis, and a Venn diagram was plotted to compare the BP terms significantly enriched to isolated adhesion complex and WCL datasets. (**c**) The -log10-transformed *P*-values for the ten highest ranking BP terms significantly enriched to the A375 WCL phosphoprotein datset, as well as two other phosphoprotein datasets collected using the WCLs of HeLa cells[23] (5192 proteins) and human embryonic stem cells[24] (hESCs; 4061 proteins). For display purposes, 'replicate' BP terms (those representing the exact same population of proteins as another BP term) were removed from panel (**c**).

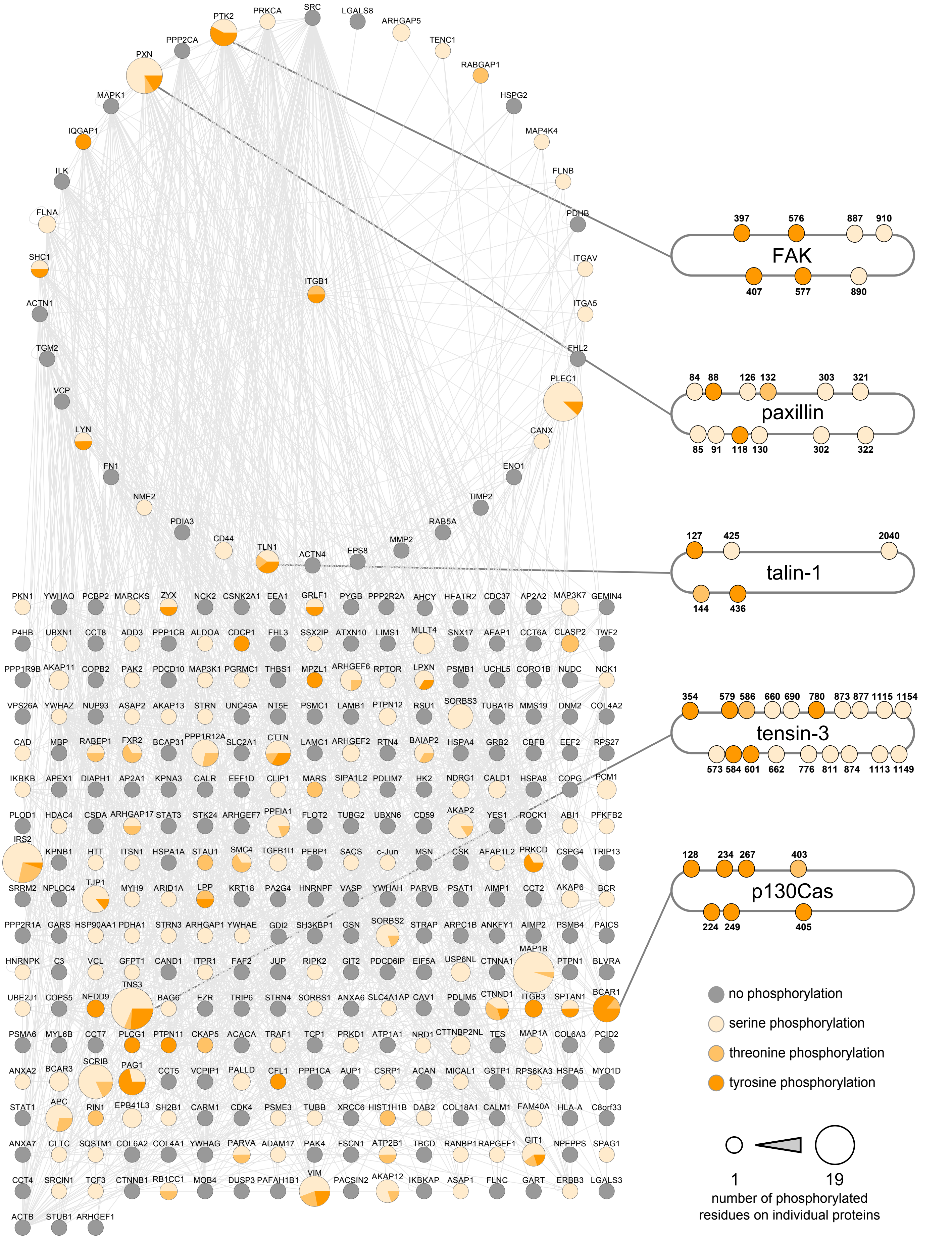

Supplementary Figure 2 Proteins and phosphoproteins within one and two binding interactions of β1 integrin. All identified proteins and phosphoproteins that were within one binding interaction (1-hop) or two binding interactions (2-hop) of β1 integrin were mapped onto a human PPI network. The 1-hop neighbourhood is displayed as a circular layout surrounding β1 integrin at the top of figure (organised anti-clockwise by connectivity). The 2-hop neighbourhood is displayed as a grid layout directly beneath the 1-hop neighbourhood. Selected phosphoproteins are displayed as larger rectangular nodes to the right of the figure, giving more detailed information on the specific phosphorylated residues identified from these proteins. Node size and colour represent different levels of phosphorylation, as described for Figure 2.

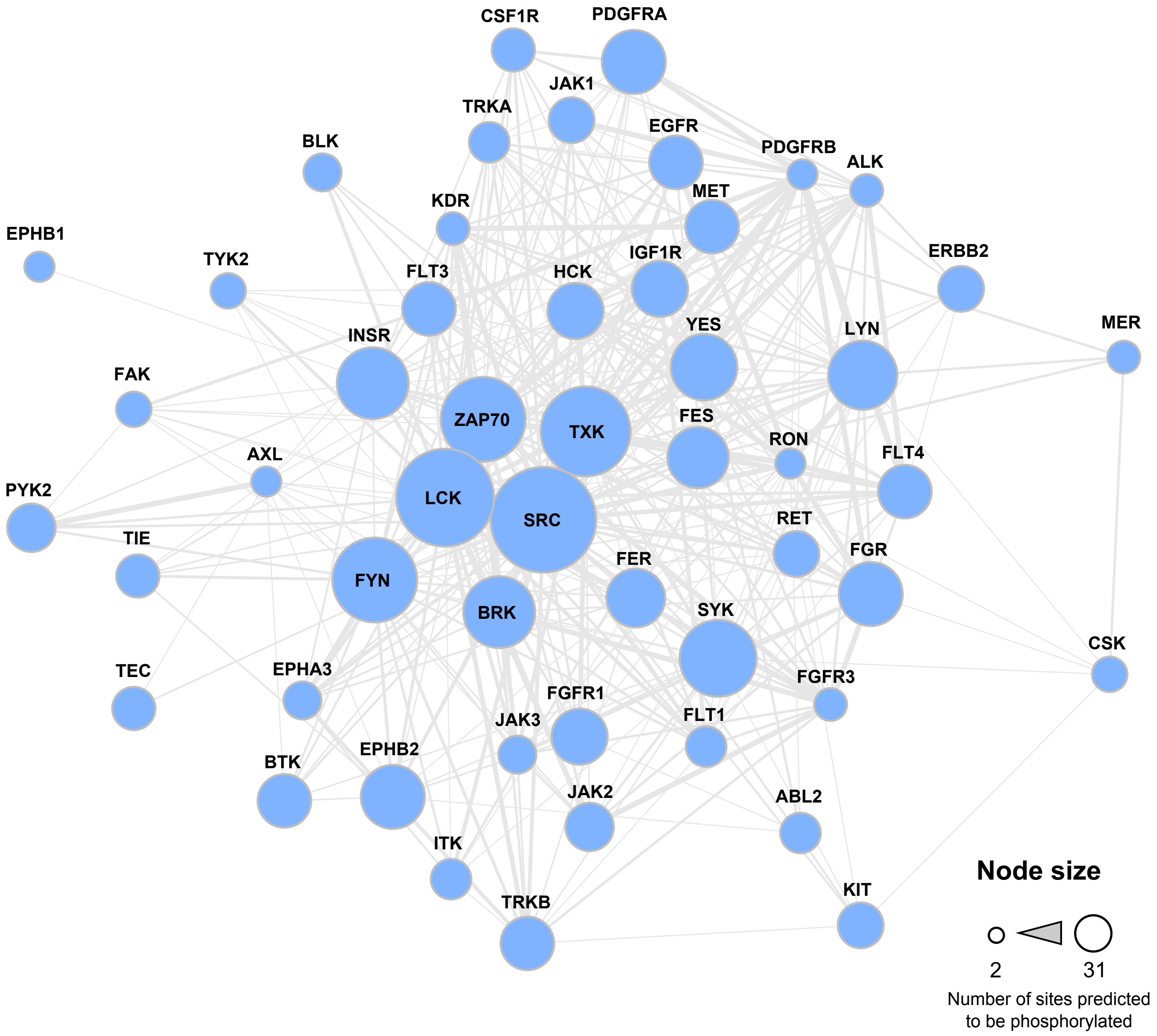

Supplementary Figure 3 Tyrosine kinases predicted to phosphorylate adhesion complex proteins. Phosphorylation sites identified by phosphoproteomic analysis of isolated adhesion complexes were searched using the kinase prediction tool GPS (version 2.1)[31]. All tyrosine kinases predicted to phosphorylate the identified phosphorylation sites are displayed. Each node represents an individual kinase or group/family of kinases. An edge connecting two nodes indicates that the corresponding kinase groups were predicted to phosphorylate at least one common residue. Nodes are clustered according to connectivity (i.e. clustered nodes were predicted to phosphorylate similar residues). Node size corresponds to the total number of adhesion complex phosphorylation sites that were predicted to be phosphorylated by the corresponding kinase.

a

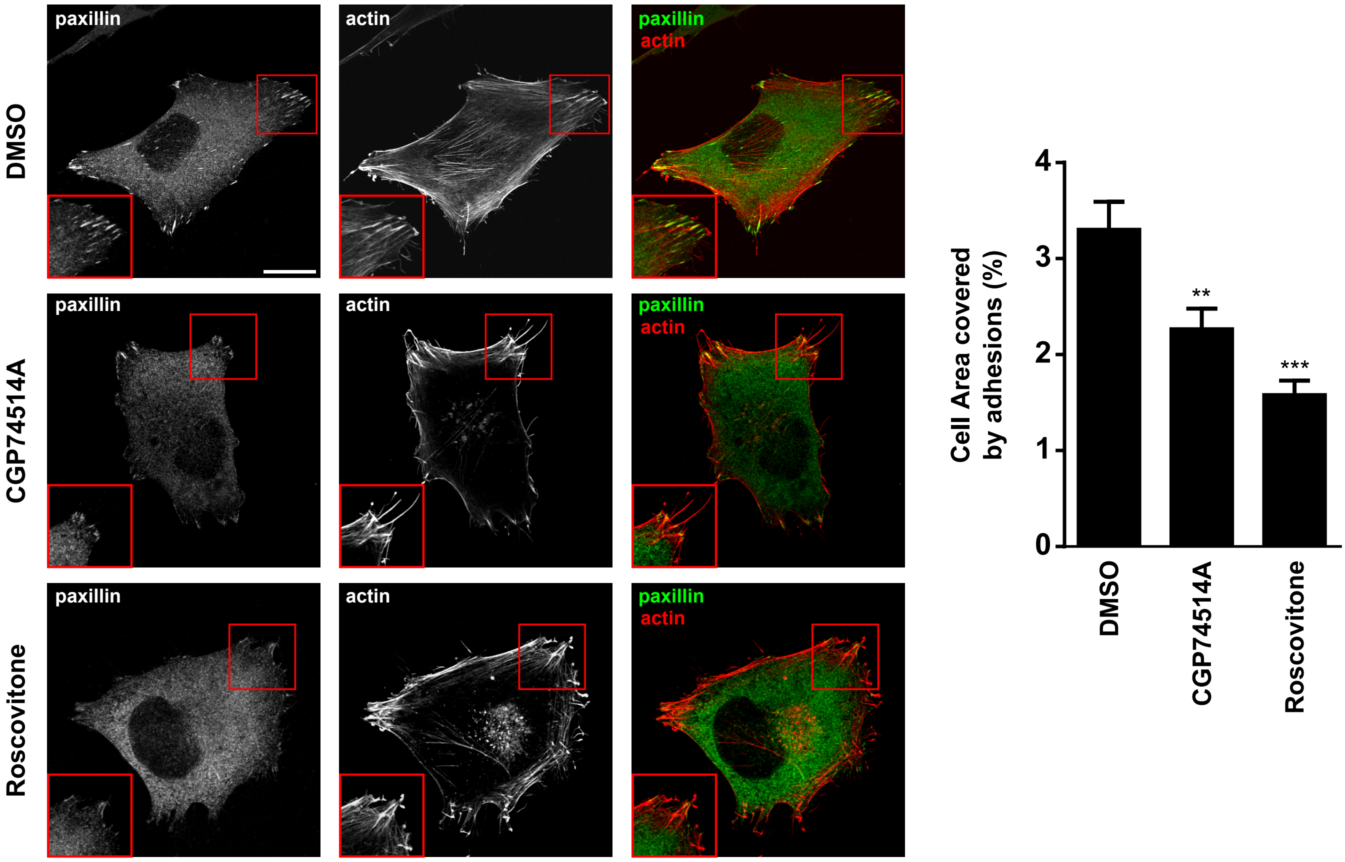

b

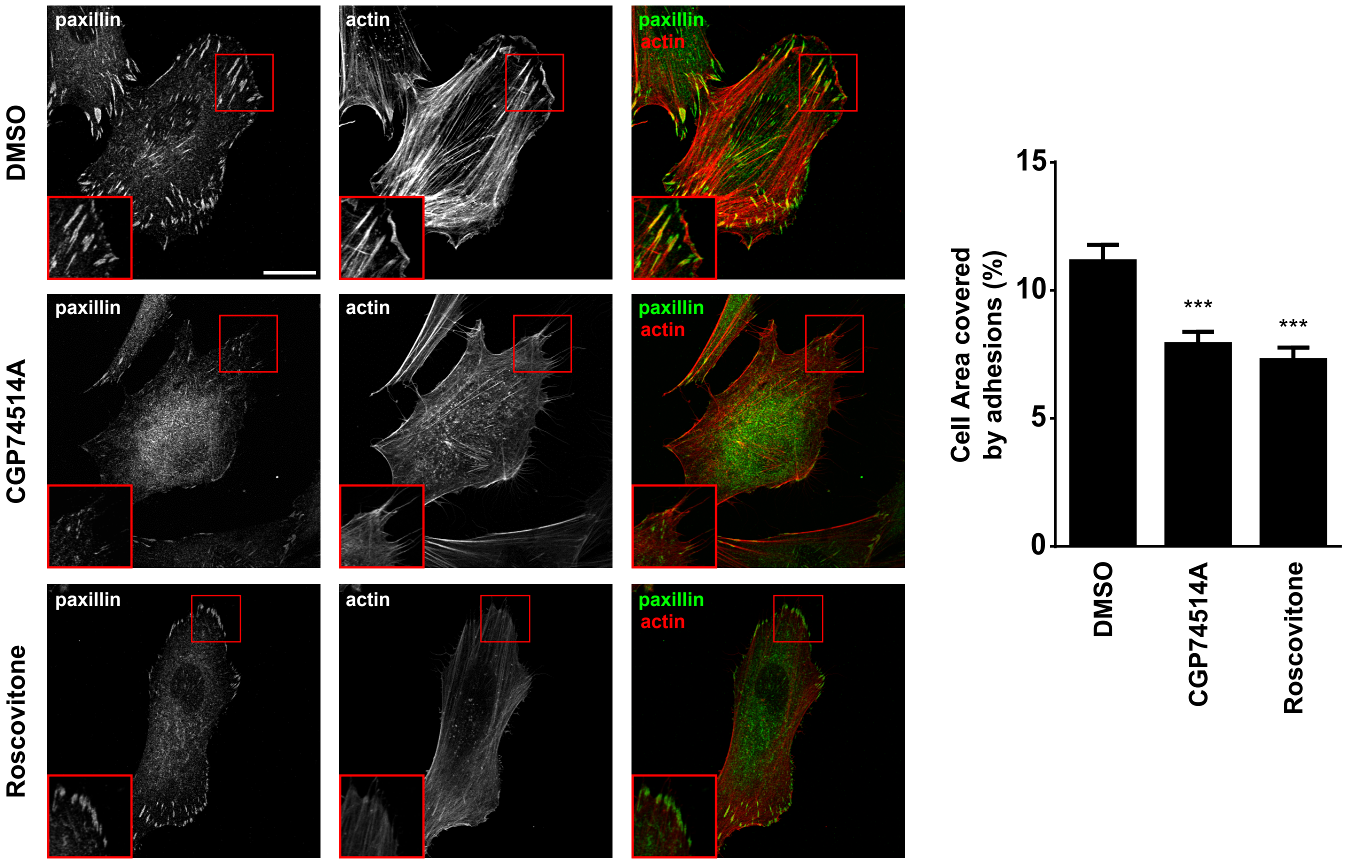

Supplementary Figure 4 Effect of alternative CDK1 inhibitors on cell adhesion. A375 cells (**a**) and human foreskin fibroblasts (HFFs; **b**) were spread on FN for 60 min before treatment with DMSO or the CDK1-specific inhibitors CGP74514A or Roscovitone. Adhesion sites were visualised by immunofluorescence staining for paxillin (green) and the actin cytoskeleton was visualised by staining with Texas Red-conjugated phalloidin (red). For both cell types, the cell area covered by adhesion sites was quantified and is displayed to the right of immunofluorescence images (n=3, quantification based on 36 cells for each cell type). Scale bar, 20 μm. Error bars represent s.e.m (** P<0.01; *** P<0.001; student's t test).

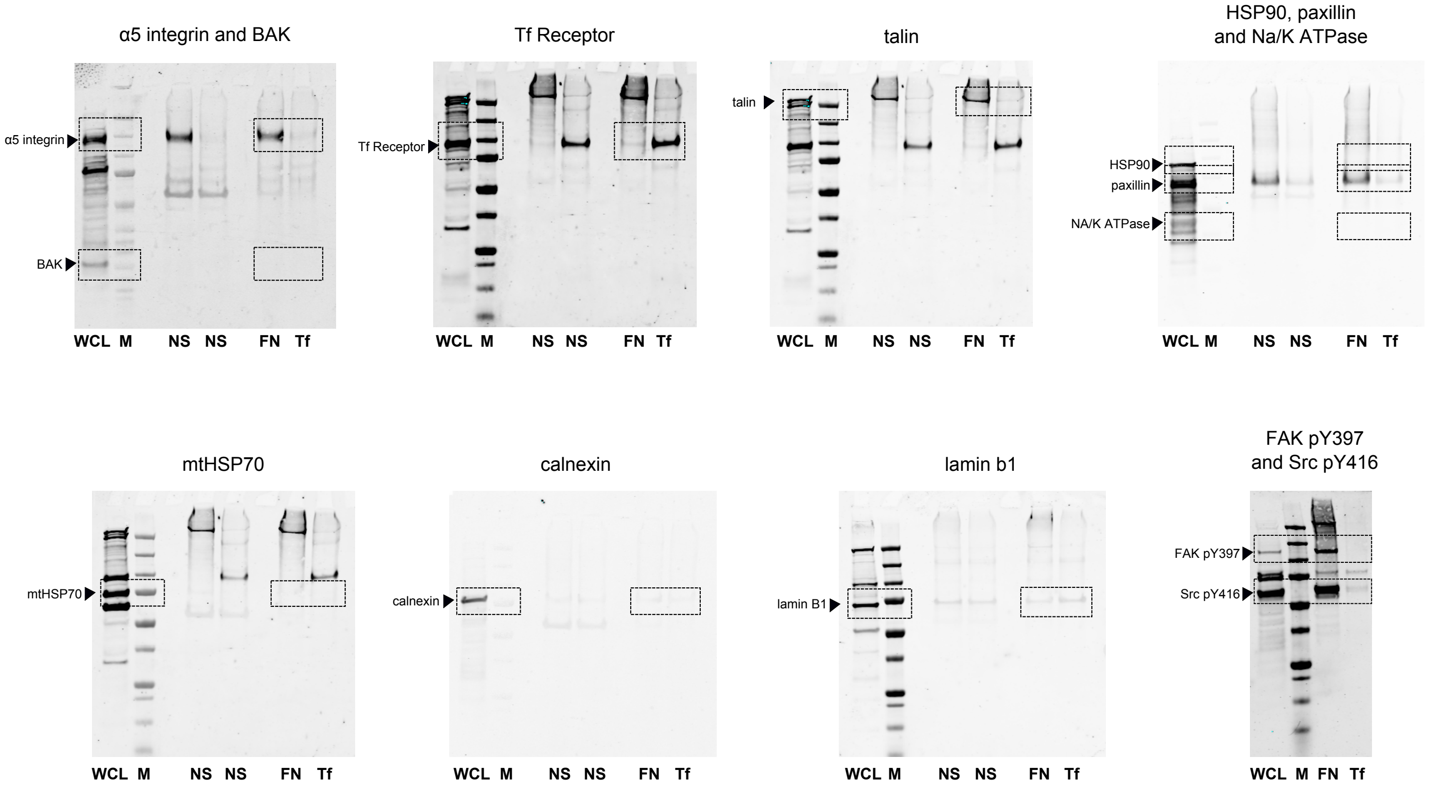

Supplementary Figure 5 Original blots for Figure 1. Dashed boxes indicate cropped regions displayed in Figure 1b. Arrows indicate the expected band position for each protein or phosphorylation site probed. M, molecular weight markers; NS, non-relevent sample; WCL, whole cell lysate

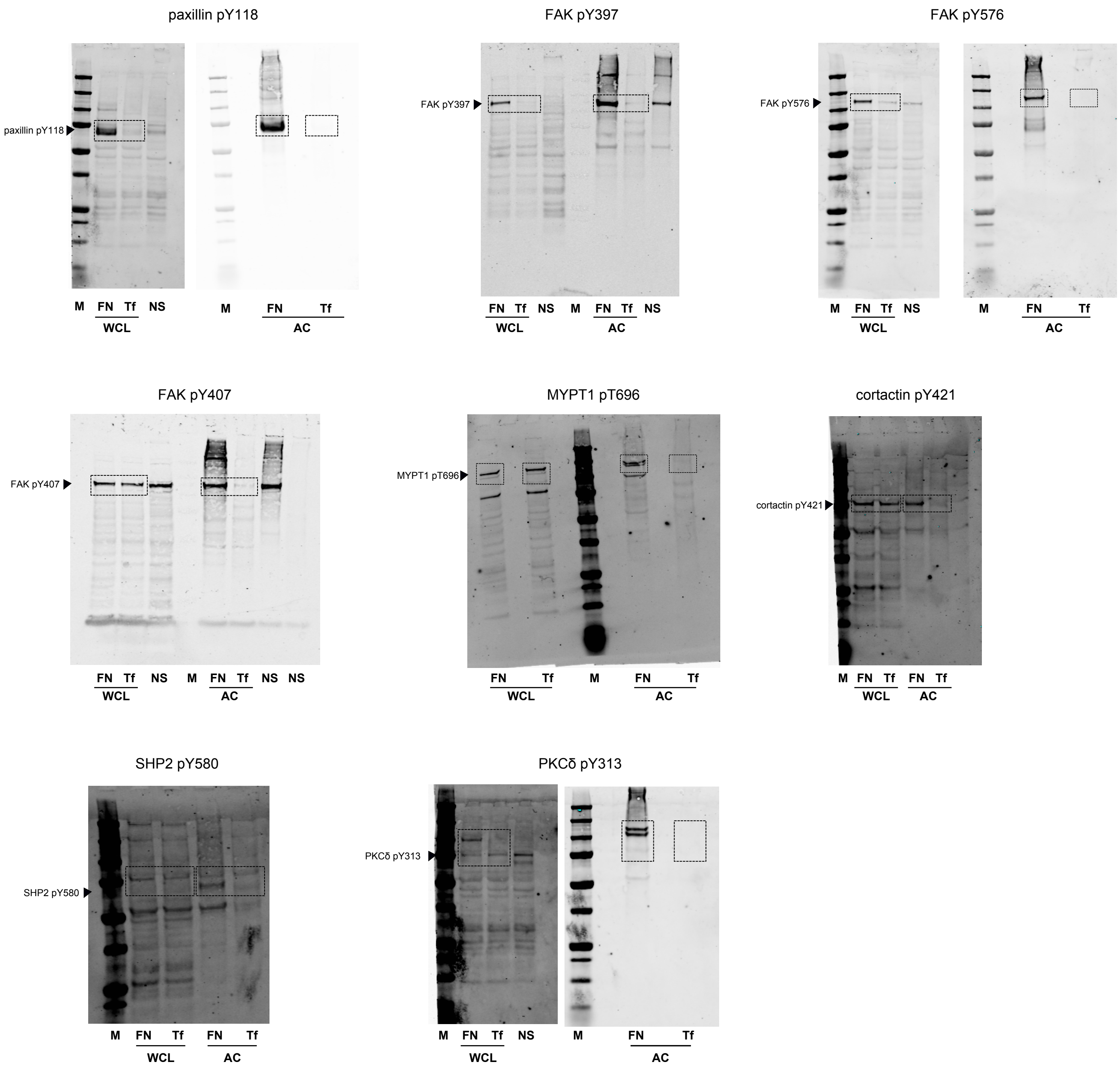

Supplementary Figure 6 Original blots for Figure 4. All phosphorylation sites that were probed for detection in both isolated adhesion complexes (Figure 4a; right hand side of each panel in this figure) and whole cell lysates (Figure 4c; left hand side of each panel in this figure) are displayed. Dashed boxes indicate cropped regions displayed in Figure 4a and c. Arrows indicate the expected band position for each phosphorylation site probed. AC, adhesion complex; M, molecular weight markers; NS, non-relevent sample; WCL, whole cell lysate
